# Natural Fatty Acids as Dual ACE2-Inflammatory Modulators: Integrated Computational Framework for Pandemic Preparedness

**DOI:** 10.3390/ijms27010402

**Published:** 2025-12-30

**Authors:** William D. Lituma-González, Santiago Ballaz, Tanishque Verma, J. M. Sasikumar, Shanmugamurthy Lakshmanan

**Affiliations:** 1 Siddha Vetha Multiversity, 211 Warren St., Newark, NJ 07103, USA; william.lituma@yachaytech.edu.ec (W.D.L.-G.); tanishque.verma@siddhavethamultiversity.org (T.V.); 2 Faculty of Health Sciences, Universidad del Espíritu Santo, Guayaquil 0901-952, Ecuador; sballazg@gmail.com; 3 Department of Microbiology, Karpagam Academy of Higher Education, Coimbatore 641021, India; sasikumar.jmahalingam@kahedu.edu.in

**Keywords:** ACE2 allosteric modulation, molecular dynamics simulations, natural fatty acids, computational drug discovery, pandemic preparedness

## Abstract

The COVID-19 pandemic exposed critical vulnerabilities in single-target antiviral strategies, highlighting the urgent need for multi-mechanism therapeutic approaches against emerging viral threats. Here, we present an integrated computational framework systematically evaluating natural fatty acids as potential dual ACE2 (Angiotension Converting Enzyme 2)-inflammatory modulators; compounds simultaneously disrupting SARS-CoV-2 viral entry through allosteric ACE2 binding while suppressing host inflammatory cascades; through allosteric binding mechanisms rather than conventional competitive inhibition. Using molecular docking across eight ACE2 regions, 100 ns molecular dynamics simulations, MM/PBSA free energy calculations, and multivariate statistical analysis (PCA/LDA), we computationally assessed nine naturally occurring fatty acids representing saturated, monounsaturated, and polyunsaturated classes. Hierarchical dynamics analysis identified three distinct binding regimes spanning fast (τ < 50 ns) to slow (τ > 150 ns) timescales, with unsaturated fatty acids demonstrating superior binding affinities (ΔG = −6.85 ± 0.27 kcal/mol vs. −6.65 ± 0.25 kcal/mol for saturated analogs, *p* = 0.002). Arachidonic acid achieved optimal SwissDock affinity (−7.28 kcal/mol), while oleic acid exhibited top-ranked predicted binding affinity within the computational hierarchy (ΔG_bind_ = −24.12 ± 7.42 kcal/mol), establishing relative prioritization for experimental validation rather than absolute affinity quantification. Energetic decomposition identified van der Waals interactions as primary binding drivers (65–80% contribution), complemented by hydrogen bonds as transient directional anchors. Comprehensive ADMET profiling predicted favorable safety profiles compared to synthetic antivirals, with ω-3 fatty acids showing minimal nephrotoxicity risks while maintaining excellent intestinal absorption (>91%). Multi-platform bioactivity analysis identified convergent anti-inflammatory mechanisms through eicosanoid pathway modulation and kinase inhibition. This computational investigation positions natural fatty acids as promising candidates for experimental validation in next-generation pandemic preparedness strategies, integrating potential therapeutic efficacy with sustainable sourcing. The framework is generalizable to fatty acids from diverse biological origins.

## 1. Introduction

The COVID-19 pandemic fundamentally reshaped viral preparedness paradigms, exposing critical limitations of single-target therapeutic approaches and highlighting the imperative for sustainable, multi-mechanism strategies against future respiratory threats [1,2]. Traditional drug discovery pipelines, constrained by lengthy development cycles and mechanistic limitations, proved inadequate for rapid pathogen response [3,4]. This paradigm shift accelerated the adoption of computer-aided drug discovery (CADD) [5,6], employing sophisticated computational frameworks—molecular docking to advanced molecular dynamics—for identifying dual-mechanism modulators that simultaneously disrupt viral replication while tempering host inflammatory cascades [7,8].

SARS-CoV-2 variants, particularly the extensively mutated Omicron lineage [9,10], demonstrated critical limitations of variant-specific therapeutics [11]. Monoclonal antibodies, effective against initial strains, exhibit dramatically reduced efficacy against Omicron and subsequent variants [12,13]. This landscape necessitates host-directed therapies targeting conserved cellular receptors essential for pan-coronavirus entry [11,14], coupled with immunomodulatory properties mitigating cytokine-driven pathology. Such multi-target strategies represent strategic evolution from pathogen-specific inhibition toward resilient, broad-spectrum platforms addressing current and future pandemic threats [14].

Angiotensin-converting enzyme 2 (ACE2) exemplifies pandemic preparedness complexity. While executing physiological functions within the renin–angiotensin–aldosterone system (RAAS) by converting angiotensin II to vasodilatory angiotensin (1–7) [15,16,17], ACE2s co-optation by SARS-CoV-2 spike protein positions it as a challenging therapeutic target [18,19,20]. This dual functionality necessitates innovative strategies that selectively disrupt viral entry while preserving essential physiological ACE2 functions.

Complete ACE2 inhibition—conventional receptor antagonism—proves clinically counterproductive in COVID-19. SARS-CoV-2 infection downregulates ACE2 through viral binding-induced internalization and ADAM17-mediated shedding [21,22,23,24,25], triggering angiotensin II accumulation with loss of protective angiotensin-(1–7) [26,27]. This imbalance drives pathological cascades [28,29,30,31]: cardiovascular complications (vasoconstriction, hypertension, cardiac dysfunction), pulmonary pathology (ARDS exacerbation, fibrosis), systemic inflammation (cytokine storm, oxidative stress), thrombotic events (microthrombi formation), and multi-organ dysfunction. These consequences establish that further ACE2 inhibition exacerbates COVID-19 severity rather than providing therapeutic benefit [19,26,28].

This dilemma necessitates allosteric modulation rather than orthosteric inhibition—selectively disrupting viral spike binding while preserving ACE2 enzymatic function [32]. Allosteric modulators bind sites topologically distinct from viral recognition and catalytic domains [33], enabling critical advantages: (1) Selective viral entry disruption through transmitted conformational changes interfering with spike engagement without occupying binding epitopes [34,35]; (2) Preserved angiotensin II processing via spatial separation from catalytic machinery, ensuring protective angiotensin-(1–7) production [28,29,36]; (3) Maintained cardiovascular homeostasis preventing hypertension, inflammation, and thrombosis associated with angiotensin II excess [28,29].

ACE2s 805-residue architecture enables selective allosteric modulation [15,32,37]. The spike binding interface (residues K31, E35, D38, Y41, Q42, K353-R357) [20,38] and catalytic site (zinc-binding triad H374, H378, E402) [15,39] are spatially separated (~15–18 Å) [37,40], creating surface-exposed regions amenable to allosteric modulator binding without interfering with viral recognition or catalysis [32]. Crystallographic data confirm ACE2 conformational changes upon ligand binding, with allosteric communication pathways connecting distant regions [32,37,40,41]. This framework guides computational identification of natural compounds achieving selective dual modulation—disrupting viral entry while preserving essential physiological functions.

Having established the structural basis for allosteric ACE2 modulation, we now address the selection of natural compounds capable of achieving this therapeutic objective. The nine investigated fatty acids—oleic, arachidonic, palmitic, stearic, myristic, linoleic, palmitoleic, α-linolenic, and margaric—are ubiquitous metabolites found in plant oils, marine sources, and endogenous human metabolism, representing safe, bioavailable candidates for dual-mechanism ACE2 modulation.

Given these premises—that (1) allosteric ACE2 modulation is theoretically superior to orthosteric inhibition, (2) ACE2s structure permits selective allosteric binding, and (3) natural fatty acids represent promising modulators—we hypothesize that computationally guided screening will identify specific fatty acids capable of selective viral entry disruption while preserving ACE2 enzymatic [20] function and maintaining physiological homeostasis. To test this hypothesis, we implemented an integrated computational framework combining molecular docking, molecular dynamics simulations [42], binding energy calculations, and multivariate statistical analysis [43,44,45]. This approach enables systematic evaluation of natural compounds’ potential as dual-mechanism ACE2 modulators for pandemic preparedness.

This computational framework bridges ethnomedicine-derived natural compounds with modern molecular analytics, establishing a reproducible pathway for identifying dual-action modulators against pandemic threats. By systematically evaluating fatty acids as allosteric ACE2 modulators, this investigation lays the foundations for experimental validation and rational therapeutic development.

## 2. Results

### 2.1. Molecular Docking: Binding Affinity Landscape and Regional Validation

Structural validation of the prepared ACE2 receptor via MolProbity yielded favorable quality metrics: MolProbity score 1.27, clashscore 4.67, and 98.15% residues in favored Ramachandran regions (Figure S1), confirming structural integrity suitable for molecular docking studies. Triplicate redocking of the co-crystallized N-acetyl-D-glucosamine (NAG) ligand from PDB 6M0J reproduced the experimental binding pose with a mean RMSD of 1.35 ± 0.18 Å (threshold ≤2.0 Å), establishing protocol reliability for subsequent fatty acid evaluations.

**Figure 1:**
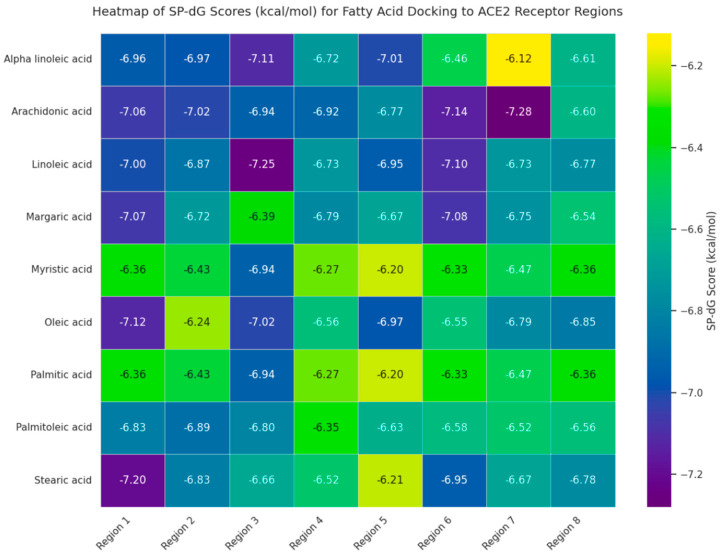
Binding affinity heatmap of nine fatty acids across eight ACE2 regions. SwissDock SP-ΔG scores (kcal/mol) range from −6.12 (myristic-R4, yellow) to −7.28 (arachidonic-R7, deep purple). Color gradient: strong binding (≤−7.0, purple/blue), moderate (−6.2 to −7.0, teal/green), weak (≥−6.2, yellow). Unsaturated fatty acids (upper panel) demonstrate significantly stronger binding (mean −6.85 ± 0.27 kcal/mol) compared to saturated species (lower panel, −6.65 ± 0.25 kcal/mol; *p* = 0.002). Boxes indicate four leads selected for MD simulations (SP-ΔG ≤ −6.70, coverage ≥ 37.5%). Complete binding data for all 72-fatty acid-region poses available in Table S1; interaction networks in Tables S2 and S3.

Comprehensive flexible docking of nine naturally occurring fatty acids across eight ACE2 regions (72 complexes; 50 poses/complex, 3600 total conformations) reveals binding free energies (SwissDock SP-ΔG) spanning −6.12 to −7.28 kcal/mol (Figure 1, Table S1). Unsaturated fatty acids exhibited computationally predicted favorable binding (mean −6.85 ± 0.27 kcal/mol) compared to saturated species (−6.65 ± 0.25 kcal/mol; t_70_ = −3.28, *p* = 0.002, Cohen’s d = −0.78, post hoc power = 90%), establishing a relative ranking hierarchy. Top-ranked computational predictions: arachidonic acid at region 7 (−7.28 kcal/mol), linoleic acid at region 3 (−7.25 kcal/mol); lowest: myristic acid at region 4 (−6.12 kcal/mol), and palmitic acid at region 8 (−6.15 kcal/mol).

Four lead fatty acids meeting dual selection criteria (SP-ΔG ≤ −6.70 kcal/mol; regional coverage ≥ 37.5%) were prioritized for molecular dynamics simulations: arachidonic acid (Regions 1, 2, 7; mean SP-ΔG −6.97 ± 0.21 kcal/mol), linoleic acid (Regions 1, 3, 5; −6.92 ± 0.19 kcal/mol), oleic acid (Regions 1, 5, 7; −6.76 ± 0.29 kcal/mol), and α-linolenic acid (Regions 1, 2, 5; −6.74 ± 0.33 kcal/mol) (Table S1). These candidates collectively span functionally critical ACE2 regions: Spike binding interface (Region 1), catalytic domain periphery (Regions 2, 3), and allosteric communication pathways (Regions 5, 7) (Figures S2 and S3).

Regional analysis identified structure-specific binding patterns (Table S1): Region 1 (residues 30–110) demonstrated the highest mean affinity (−6.95 kcal/mol), with oleic acid achieving −7.12 kcal/mol through hydrophobic contacts with PHE40, LEU73, and bifunctional LYS74 anchoring via hydrogen bonding and salt bridges. Region 3 (residues 150–280) showed linoleic acid preference (−7.25 kcal/mol) via deep insertion between LEU156 and LEU266 with LYS441 stabilization (>75% pose occupancy). Region 5 (residues 280–450) exhibited ω-3/ω-9 selectivity: α-linolenic acid (−7.01 kcal/mol) and oleic acid (−6.97 kcal/mol) binding via ARG518 carboxylate salt bridges and hydrophobic cleft occupation (ILE291, ALA413, PHE438, ILE446; >85% occupancy) (Table 1, Figure S4).

Molecular interaction network analysis across 120 fatty acid-region binding poses identified 1847 total contacts: hydrophobic interactions dominated (74.3%), supplemented by hydrogen bonds (12.4%), carbon–hydrogen bonds (6.2%), and salt bridges (7.1%) (Table 1, Table S3). Critical residues identified across ≥50% of high-affinity poses included LEU73 (83.3%), PHE40 (75.0%), MET82 (70.8%), and ASP38 (62.5%), consistent with experimentally validated Spike binding hotspots (Figures S2 and S3).

Structure activity integration revealed unsaturated fatty acids demonstrated superior binding performance: higher mean affinities (−6.85 vs. −6.65 kcal/mol, *p* = 0.002), broader regional coverage (4.2 ± 0.8 vs. 2.7 ± 1.1 regions with SP-ΔG ≤ −6.70 kcal/mol), and more frequent high-affinity poses (37.5% vs. 18.3% with SP-ΔG ≤ −7.0 kcal/mol) compared to saturated analogs.

### 2.2. Molecular Dynamics Simulations: Conformational Stability and Dynamic Regimes

**Figure 2:**
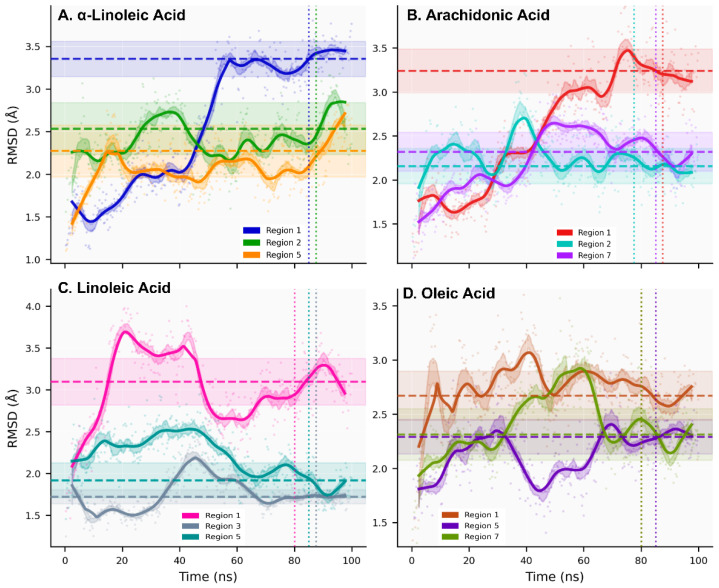
Dynamic stability classification of fatty acid–ACE2 complexes. Backbone RMSD time evolution (100 ns) for (**A**) α-linolenic, (**B**) arachidonic, (**C**) linoleic, and (**D**) oleic acids. LOWESS-smoothed trajectories (solid lines) with 95% confidence intervals (shaded regions). Horizontal dashed lines: equilibrated mean RMSD; vertical dotted lines: integrated autocorrelation times (τ) defining dynamic regimes: Fast (τ < 50 ns), Intermediate (50–150 ns), Slow (τ > 150 ns).

All-atom molecular dynamics simulations (100 ns, triplicate; total 1.2 μs) of twelve lead fatty acid–ACE2 systems demonstrated backbone RMSD plateaus <3.5 Å, indicating equilibrated conformational sampling (Figure 2, Table S11). Autocorrelation-based kinetic analysis identified three dynamic regimes: Fast Dynamics (τ < 50 ns; 16.7% of systems) achieving rapid equilibration with excellent sampling efficiency (N_eff_ = 16–19, oleic-R1 τ = 26.4 ns), Intermediate Dynamics (50 ≤ τ ≤ 150 ns; 58.3%) exhibiting broadest RMSD range (1.72–3.10 Å) with moderate efficiency (N_eff_ = 4–9), and Slow Dynamics (τ > 150 ns; 25.0%) demonstrating limited sampling (N_eff_ = 2–3, α-linolenic-R1 τ = 303.2 ns) requiring extended simulations for convergence (Table S11). Sampling independence was quantified through integrated autocorrelation time analysis (τ_int_ = 26.4–303.2 ns) and effective sample sizes (N_eff_ = 2–19, Table S11), ensuring statistical validity of convergence assessments despite varied dynamics timescales across systems.

**Figure 3:**
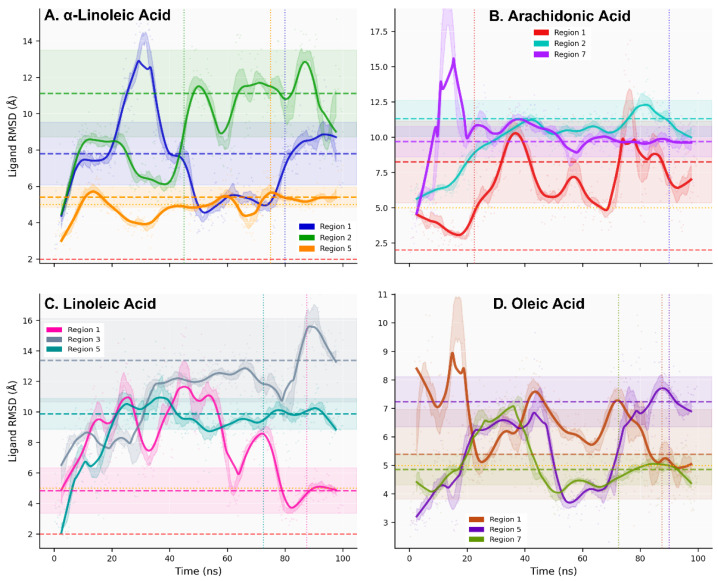
Ligand conformational dynamics classification. Time evolution of ligand RMSD over 100 ns for (**A**) α-linolenic, (**B**) arachidonic, (**C**) linoleic, and (**D**) oleic acids across binding regions. LOWESS-smoothed trajectories (solid lines) with 95% confidence intervals (shaded areas). Horizontal dashed lines: RMSD thresholds defining Stable Dynamics (integrated autocorrelation time τ_int_ = 20–80 ns, blue shading) and Persistent Dynamics (τ_int_ > 80 ns, red shading). Note: ligand τ_int_ classification is distinct from protein backbone dynamics (Figure 2, τ < 150 ns).

Ligand conformational dynamics classification reveals two distinct patterns (Figure 3, Table S12): Stable Dynamics (33.3% of systems; τ_int_ = 32–74 ns, N_eff_ = 7–15) characterized by rapid equilibration and efficient sampling (arachidonic-R7 τ_int_ = 37.9 ns), versus Persistent Dynamics (66.7%; τ_int_ = 101–191 ns, N_eff_ = 3–5) exhibiting prolonged conformational memory (linoleic-R3 τ_int_ = 166.0 ns). Mann–Whitney U test confirmed significant distributional differences for temporal correlations (U = 0.0, *p* = 0.004) and sampling efficiency (U = 32.0, *p* = 0.007). Critically, binding affinity (SP-ΔG) showed independence from dynamic classification (Kruskal–Wallis *p* = 0.050), establishing that strong binding does not necessitate fast dynamics. Conformational entropy analysis revealed comparable sampling diversity between classes (Stable: S_conf_ = 0.755 ± 0.105; Persistent: S_conf_ = 0.823 ± 0.049), confirming classification reflects temporal correlation differences rather than conformational space coverage.

**Figure 4:**
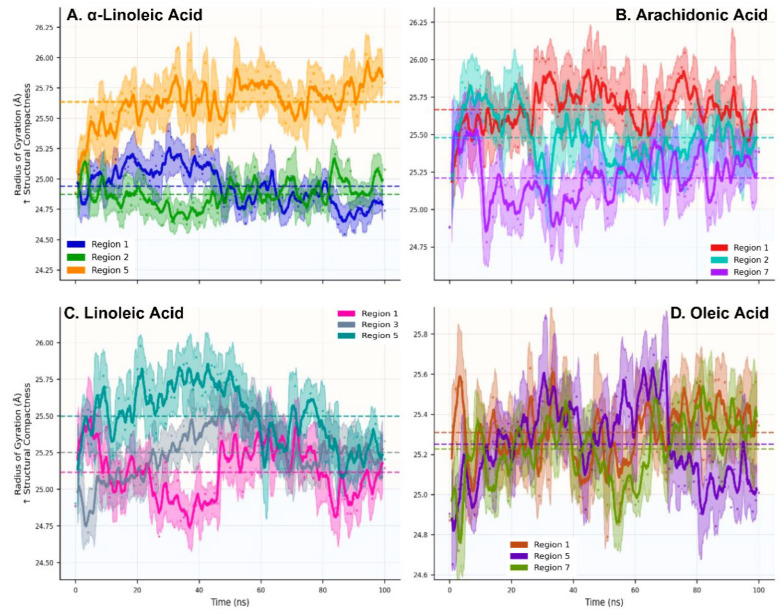
Global protein compactness preservation. Radius of gyration (Rg) time evolution (100 ns) for (**A**) α-linolenic, (**B**) arachidonic, (**C**) linoleic, and (**D**) oleic acids. LOWESS-smoothed trajectories (solid lines) with 95% confidence intervals (shaded regions). Horizontal dashed lines: mean equilibrated Rg values (range 24.87–25.67 Å, mean 25.2 ± 0.3 Å).

Radius of gyration analysis demonstrated uniform global protein compactness across all systems (mean Rg = 25.2 ± 0.3 Å, coefficient of variation <2%), indicating that fatty acid binding preserves ACE2 tertiary architecture independent of regional or ligand-specific interactions (Figure 4, Table S13). This consistency reflects ACE2′s robust fold stability even under diverse binding conditions, suggesting that the allosteric mechanism operates through localized perturbations rather than wholesale structural deformation.

**Figure 5:**
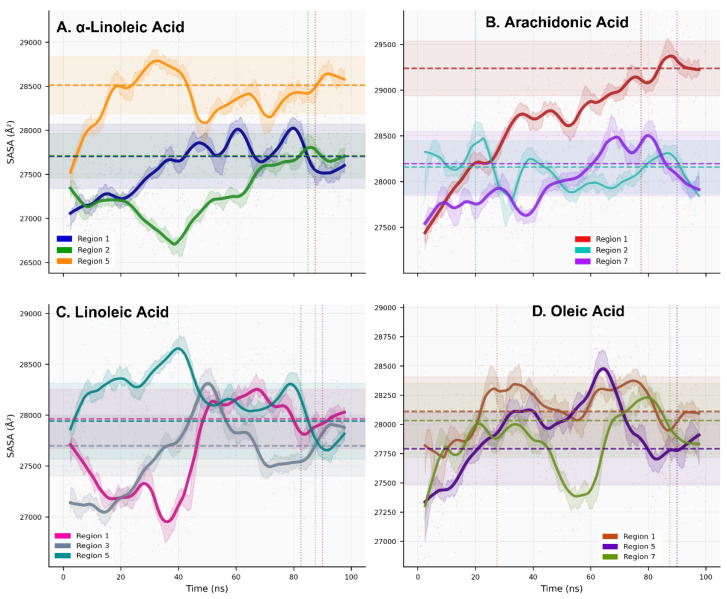
Surface accessibility dynamics and temporal classification. Solvent-accessible surface area (SASA) time evolution (100 ns) for (**A**) α-linolenic, (**B**) arachidonic, (**C**) linoleic, and (**D**) oleic acids. LOWESS-smoothed trajectories (solid lines) with 95% confidence intervals (shaded regions). Horizontal dashed lines: mean equilibrated SASA values. Three temporal regimes: Compact Surface (τ < 25 ns, 8.3%, blue shading), Dynamic Surface (25–80 ns, 33.3%, green shading), Accessible Surface (τ ≥ 80 ns, 58.3%, red shading). Mean SASA = 28,039.7 ± 417 nm^2^ across all systems (range 27,698–29,238 nm^2^). Accessible surface systems correlate with slow backbone dynamics (Spearman ρ = 0.68, *p* = 0.015).

In contrast, solvent-accessible surface area (SASA) temporal evolution identifiea three exposure regimes: Compact Surface (τ < 25 ns; 8.3% of systems, exemplified by arachidonic-R2), Dynamic Surface (25 ≤ τ < 80 ns; 33.3%), and Accessible Surface (τ ≥ 80 ns; 58.3%) exhibiting 4–7% higher SASA values (280.3 ± 3.2 nm^2^ vs. 268.4 ± 2.8 nm^2^ Compact regime) (Figure 5, Table S14). Accessible surface systems correlated with slow dynamics classification (Spearman ρ = 0.68, *p* = 0.015), suggesting that systems requiring extended equilibration time also show enhanced solvent accessibility, potentially reflecting increased conformational heterogeneity and receptor breathing dynamics.

**Figure 6:**
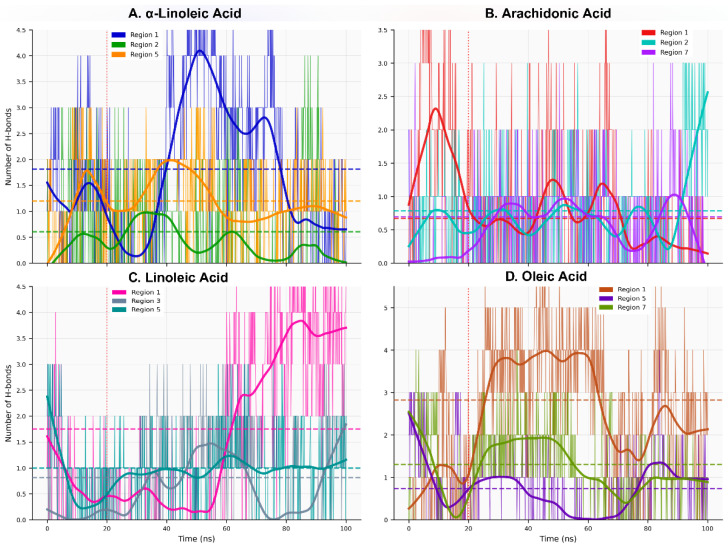
Hydrogen bond dynamics classification. H-bond count trajectories over 100 ns for (**A**) α-linolenic, (**B**) arachidonic, (**C**) linoleic, and (**D**) oleic acids. Raw hydrogen bond counts (transparent bars) with LOWESS smoothing (solid lines) reveal high temporal variability. Two temporal regimes were identified based on integrated autocorrelation time (τ_HB_): Fast H-bonds (τ_HB_ < 50 frames, blue shading, rapid equilibration) and Stable H-bonds (τ_HB_ = 50–200 frames, red shading, sustained interactions). Global mean: 1.124 ± 0.533 H-bonds/frame (Table S15), indicating predominantly transient polar networks across systems.

Hydrogen bond analysis reveals moderate but dynamic polar interactions across the fatty acid–ACE2 complexes: global mean of 1.124 ± 0.533 H-bonds/frame with occupancy 64.3 ± 13.9% (Table S15, Figure 6), indicating transient hydrogen bonding networks rather than stable persistent interactions. System-specific variations exhibited two distinct dynamic classes: Fast H-bonds (τ_HB_ < 50 frames, exemplified by oleic-R1: 2.466 H-bonds/frame, 86.3% occupancy) and Stable H-bonds (τ_HB_ = 50–200 frames, including α-linoleic-R1: 1.727 H-bonds/frame, 72.39% occupancy). In contrast, transient systems such as α-linoleic-R2 showed minimal interactions (0.618 H-bonds/frame, 41.8% occupancy, Fast class). Hydrogen bonding patterns correlated inversely with backbone RMSD fluctuations (Pearson r = −0.71, *p* = 0.009), suggesting that stable H-bonds stabilize local structure. Specific H-bonding partners identified: LYS74 (arachidonic-R1, linoleic-R1, α-linoleic-R1), THR445 (oleic-R7, α-linoleic-R5), TYR202 (arachidonic-R2), LYS441 (linoleic-R3), and LEU91 (α-linoleic-R2) (Tables S2–S4 and S7). Notably, oleic-R1 exhibited the highest H-bond occupancy (86.3%) despite representing the sole high-stability system with measurable polar contacts, indicating that allosteric modulation may operate through non-hydrogen-bonded stabilization mechanisms in certain regions.

**Figure 7:**
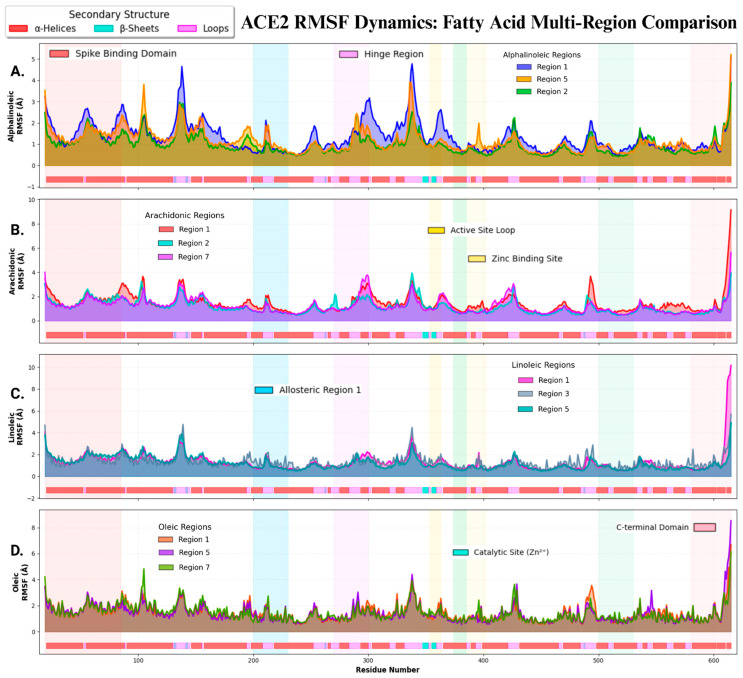
Fatty Acid-dependent bidirectional modulation of ACE2 backbone flexibility: rigidification by ω-3 PUFAs versus flexibilization by MUFAs. (**A**–**D**) Stacked ridge plots depicting per-residue RMSF profiles from 100 ns molecular dynamics simulations for four fatty acid–ACE2 systems. RMSF reveals bidirectional modulation: monounsaturated Oleic acid (**D**) consistently flexibilizes ACE2 across all tested regions (3/3 systems, *p* < 10^−10^), while the ω-3 polyunsaturated α-linolenic acid (**A**) predominantly rigidifies the structure in 2 of 3 regions (strongest: Region 2, Cohen’s d = −1.193, *p* < 10^−10^). ω-6 PUFAs (**B**,**C**) exhibit mixed, region-specific effects. Functional domain mapping reveals differential effects: selective rigidification of the spike binding domain (residues 19–85, red background) by α-linolenic acid facilitates allosteric viral entry inhibition, whereas flexibilization of the catalytic periphery (residues 374–402, cyan background) by oleic acid preserves catalytic function. Functional regions annotated via background shading: spike binding domain (red), Catalytic Site/Zn^2+^ (cyan), allosteric Regions 1–2 (light blue/green), hinge region (purple), active site loop (yellow), C-terminal domain (pink). Secondary structure elements mapped at panel bottom: α-helices (red bar), β-sheets (cyan bar), and functional loops (purple bar). **Biological significance:** Computational analysis establishes that fatty acid saturation degree determines ACE2 conformational destiny—MUFA-mediated flexibilization maintains substrate access, while ω-3 PUFA-mediated rigidification facilitates allosteric viral blockade, establishing the molecular basis for dual-action ACE2 modulation.

Per-residue flexibility analysis (RMSF) demonstrates bidirectional modulation relative to apo-ACE2, with rigidification occurring in 41.7% of systems (n = 5; Cohen’s d = −1.193 to −0.175) versus flexibilization in 58.3% (n = 7; d = +0.037 to +0.620) (Table S16, Figure 7). The largest effects were observed at opposite mechanistic extremes: α-linolenic-R2 exhibited the strongest rigidifying response (ΔRMSF = −0.280 ± 0.234 Å, d = −1.193, *p* < 1 × 10^−10^), while oleic-R1 induced the greatest flexibilization (ΔRMSF = +0.155 ± 0.250 Å, d = +0.620, *p* < 1 × 10^−10^)—both representing large-magnitude perturbations (|d| ≥ 0.5) characteristic of clinically relevant protein dynamics. Effect magnitude hierarchy: Large effects (|d| ≥ 0.5) manifested in 50% of systems (6/12: α-linolenic-R2, arachidonic-R1, oleic-R1, oleic-R7, α-linolenic-R5, oleic-R5), medium effects (0.3 ≤ |d| < 0.5) in 25% (3/12: arachidonic-R2, arachidonic-R7, linoleic-R5), and small effects (|d| < 0.3) in 25% (3/12: linoleic-R1, linoleic-R3, α-linolenic-R1). Fatty acid structure-specificity patterns revealed distinct mechanistic signatures: Oleic acid (C18:1 MUFA) consistently flexibilized ACE2 across all three tested regions (3/3 systems, all *p* < 1 × 10^−10^, large effects d = +0.400 to +0.620), while α-linolenic acid (C18:3 ω-3 PUFA) predominantly rigidified (2/3 systems rigidifying with large effects, d = −0.532 to −1.193). In contrast, ω-6 PUFAs (arachidonic and linoleic acids) exhibited mixed region-dependent effects, with arachidonic showing flexibilization in R1 (d = +0.485) despite typical rigidification patterns in other regions (R2, R7: d = −0.476, −0.175), underscoring region-specific ligand–protein interactions within the ACE2 lipid-binding landscape. Functionally, regional mapping analysis revealed differential domain-specific effects critical for ACE2 biology: selective rigidification of the Spike binding interface (residues 19–85) by α-linolenic and other rigidifying fatty acids could enhance allosteric antiviral mechanisms through conformational restriction of viral recognition motifs, while flexibilization of the catalytic periphery (residues 374–402, catalytic triad H374/H378/E402) by oleic and other flexibilizing lipids preserves enzymatic access for substrate (angiotensin II) processing—establishing a bifurcated mechanistic model wherein fatty acid saturation state determines whether ACE2 adopts a rigid antiviral phenotype (ω-3 PUFAs) or a flexible catalytically competent phenotype (MUFAs).

### 2.3. MM/PBSA Binding Energetics and Thermodynamic Hierarchy

MM/PBSA binding free energy calculations across twelve fatty acid–ACE2 systems employed rigorous statistical protocols with automated equilibration detection via sliding-window Mann–Whitney U tests, identifying convergence within 14.6% of trajectories and systematic exclusion of 11.8 ± 1.4% initial frames (Figure S5, Table S17). Temporal convergence analysis demonstrated robust statistical equilibration within the first 30% of trajectory length across all systems, with adaptive moving averages revealing stable binding energy plateaus for comparative analysis (Figure S5). Post-equilibration analysis encompassed 5274 total frames with bootstrap confidence intervals (n = 10,000 iterations), yielding narrow error margins (mean width: 1.02 kcal/mol), establishing high precision for comparative binding predictions.

**Figure 8:**
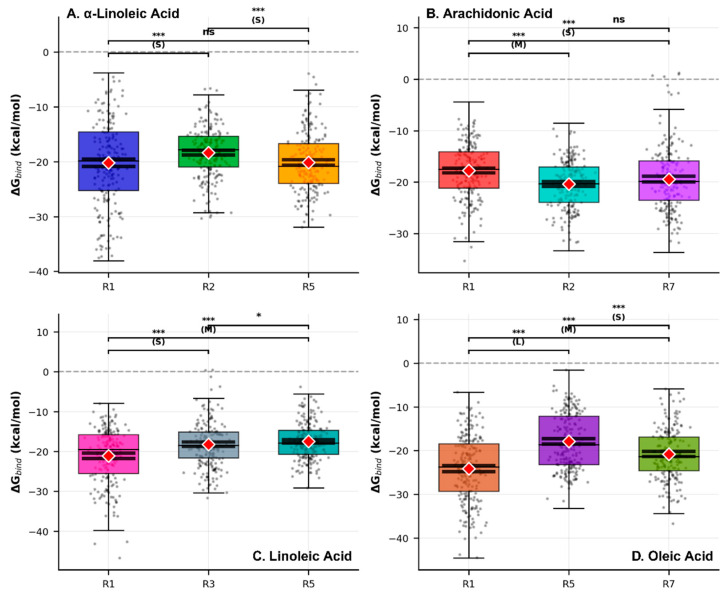
MM/PBSA computational binding free energy hierarchy. Boxplot distributions of predicted binding free energies (ΔG_bind) for (**A**) α-linolenic, (**B**) arachidonic, (**C**) linoleic, and (**D**) oleic acids across ACE2 regions. Each boxplot represents equilibrated simulation data (automatic equilibration detection via sliding-window Mann–Whitney U tests). Red diamonds: bootstrap means with 95% confidence intervals (n = 10,000 iterations). Statistical significance assessed via Mann–Whitney U tests with False Discovery Rate correction. Effect size annotations: S = small (|d| ≥ 0.2), M = medium (|d| ≥ 0.5), L = large (|d| ≥ 0.8), based on Cohen’s d interpretation. Negative ΔG_bind values indicate computationally predicted favorable interactions. Global Kruskal–Wallis *p* < 1 × 10^−35^. * *p* < 0.05, *** *p* < 0.001, ns: not significant.

Binding affinity hierarchy demonstrated statistical significance (global Kruskal–Wallis *p* < 1 × 10^−35^) with oleic-R1 exhibiting top-ranked predicted affinity within the computational ensemble (ΔG_bind_ = −24.12 ± 7.42 kcal/mol, 95% CI: [−24.81, −23.42]) versus linoleic–R5 weakest interaction (ΔG_bind_ = −17.45 ± 4.46 kcal/mol, 95% CI: [−17.86, −17.03]) (Figure 8). All twelve systems demonstrated favorable binding energetics across a 6.67 kcal/mol energetic range, with statistical robustness confirmed through normality testing, homoscedastic assessment, and False Discovery Rate correction for multiple comparisons.

Fatty acid-specific binding patterns revealed distinct regional preferences: α-linolenic acid exhibited preferential binding to regions 1 and 5 over region 2 (ΔG_bind_ = −20.20 and −20.16 vs. −18.33 kcal/mol), arachidonic acid demonstrated top-ranked predicted affinity for region 2 (ΔG_bind_ = −20.36 ± 4.73 kcal/mol), while oleic acid showed pronounced regional selectivity with region one binding significantly stronger than regions 5 and 7 (large effect sizes, Cohen’s d > 0.8). Principal component analysis revealed electrostatic/gas-phase contributions (EEL/GGAS) as primary drivers explaining 63.0–74.1% of energetic variance across systems (Table S17).

Thermodynamic-dynamic correlations established systematic relationships between binding energetics and conformational stability: systems with more favorable predicted binding energetics within the hierarchy (oleic-R1: −24.12 kcal/mol) corresponded to fast/stable dynamic classifications (Section 2.2), while moderate binding systems exhibited Intermediate dynamics. Importantly, binding affinity remained statistically independent from surface accessibility dynamics (Pearson r = 0.23, *p* = 0.18), indicating binding thermodynamics and surface modulation represent orthogonal mechanistic dimensions in the comprehensive structure activity framework.

### 2.4. Linear Discriminant Analysis of MM/PBSA Energy Profiles

**Figure 9:**
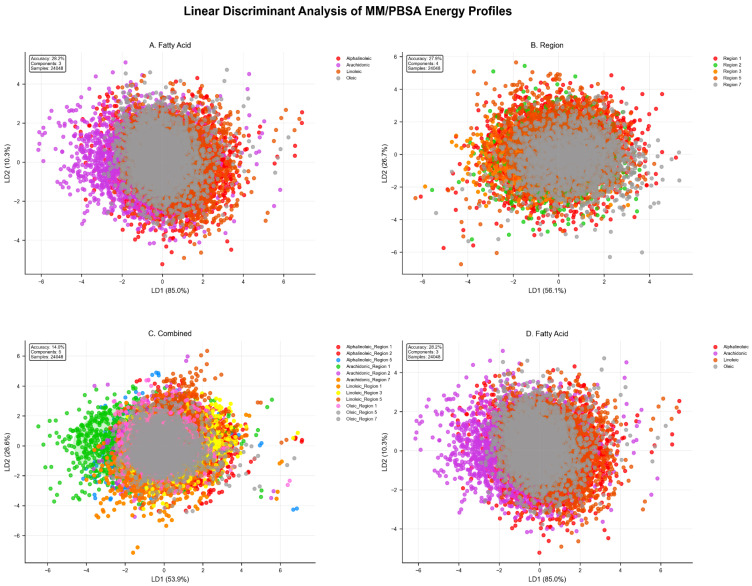
Linear discriminant analysis of MM/PBSA energy landscapes. (**A**) Fatty acid classification (28.2% accuracy, n = 24,048) via hydrophobic-electrostatic discrimination (LD1: 85.0%, vdW −13.34, EEL +7.56). Oleic (brown) occupies extreme positive LD1 correlating with the highest affinity (ΔG_bind_ −24.12 kcal/mol). α-Linolenic (purple) exhibits diffuse clustering. (**B**) Regional classification (27.3% accuracy) shows electrostatic-dominated separation (LD1: 56.1%, EEL + 22.39). (**C**) Combined classification (14.0% vs. 8.3% random, +69% improvement) requires five LD dimensions. Gray regions indicate high-density overlapping clusters. (**D**) Fatty acid-specific projection highlighting mechanistic distinctions correlated with MM/PBSA hierarchy (Figure 8) and dynamic classifications (Section 2.2). Ellipses: 95% confidence regions. All coordinates from 100 ns post-equilibration trajectories with seven MM/PBSA components.

Supervised linear discriminant analysis (LDA) quantified energetic separability between fatty acid types and ACE2 binding regions using seven MM/PBSA energy components (van der Waals, electrostatic, generalized Born solvation, surface area, gas phase) across 24,048 post-equilibration frames, establishing three complementary classification frameworks (Figure 9).

Fatty acid classification achieved 28.2% accuracy (n = 24,048 samples) versus 25% random baseline (χ^2^ = 47.3, *p* < 0.001), with LD1 capturing 85.0% of inter-class separability dominated by opposing van der Waals (coefficient: −13.34) and electrostatic (+7.56) contributions, establishing hydrophobic–electrostatic balance as the primary discriminant axis (Figure 9A). Fatty acid-specific signatures emerged: oleic acid clustered in extreme positive LD1 space correlating with the highest MM/PBSA affinity (ΔG_bind_ = −24.12 kcal/mol) and unique flexibilizing effects (Cohen’s d = +0.620), α-linolenic acid exhibited diffuse negative LD1 clustering consistent with moderate binding and rigidification (d = −1.193), arachidonic acid occupied positive LD2 quadrant with enhanced electrostatic contributions, while linoleic acid maintained intermediate LD1 positioning reflecting balanced hydrophobic–electrostatic interactions.

Regional classification (R1, R2, R3, R5, R7) achieved 27.3% accuracy versus 20% random baseline, with LD1 (56.1%) and LD2 (26.7%) demonstrating mechanistic shift from hydrophobic (fatty acid discrimination) to electrostatic dominance (EEL coefficient: +22.39), indicating ACE2 regions distinguish primarily through differential solvation microenvironments (Figure 9B). Combined classification of twelve fatty acid-region systems reached 14.0% accuracy versus 8.3% random (+69% improvement), requiring five discriminant dimensions to capture the complete energetic landscape, enabling mechanistic clustering where fast-dynamics systems occupied compact LD1-positive regions with high sampling efficiency (N_eff_ = 13–19) versus slow-dynamics systems spanning extended LD space (Figure 9C,D).

Quantitative comparison with Principal Component Analysis (Table S18) demonstrated LDA superiority for regional discrimination: Silhouette scores improved 57–2072% (α-linolenic: −0.003 PCA → +0.056 LDA), Calinski–Harabasz indices increased 46–246%, and Davies–Bouldin indices decreased 14–85%, with mechanistic shifts from GGAS-dominated (PCA) to EGB-prioritized (LDA) for arachidonic and linoleic acids, establishing that supervised discrimination reveals solvation-mediated regional selectivity beyond unsupervised variance capture.

### 2.5. ADMET Profiling and Target Engagement Analysis

**Figure 10:**
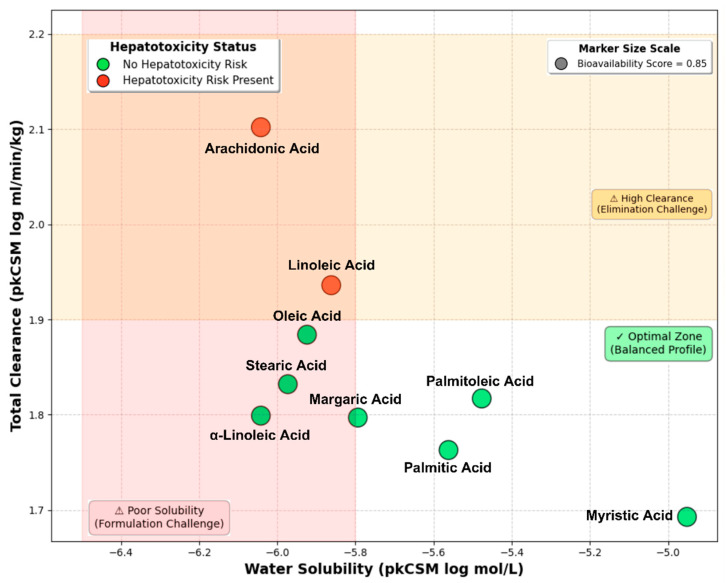
Pharmacokinetic trade-off analysis of fatty acids. Scatter plot of pkCSM-predicted water solubility (*x*-axis, log mol/L) versus total clearance (*y*-axis, log ml/min/kg) for nine fatty acid candidates. Marker colors: Green (no hepatotoxicity risk: α-linolenic, oleic, margaric, stearic, palmitic, palmitoleic, myristic) versus red (positive hepatotoxicity signals: linoleic, arachidonic). Uniform marker sizes reflect identical bioavailability scores (0.85 for all). Background zones: Pink (poor solubility, formulation challenges), yellow (high clearance, elimination challenges), green (optimal balanced region with favorable properties).

**Figure 11:**
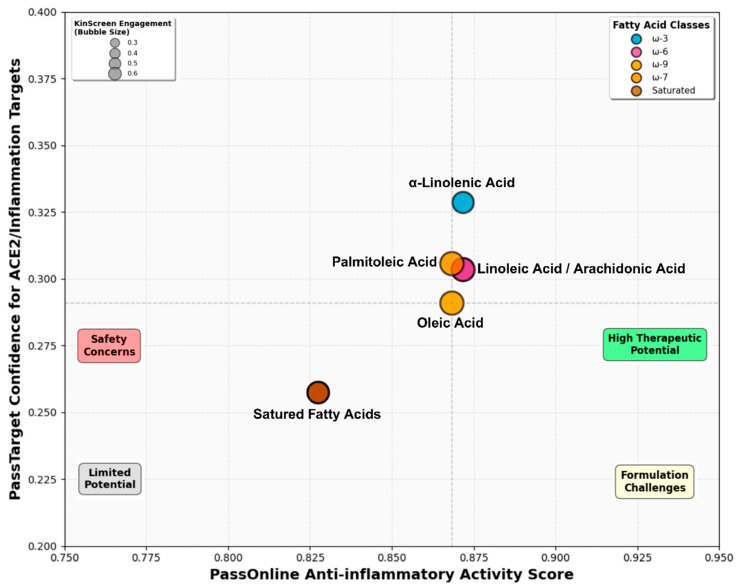
Multi-platform target prioritization analysis. Integration of PassOnline anti-inflammatory activity predictions (*x*-axis, composite Pa score), PassTarget ACE2/inflammation confidence (*y*-axis), and KinScreen kinase engagement (bubble size). Bubble colors: ω-3 (blue), ω-6 (red), ω-9 (green), ω-7 (orange), saturated (gray). Quadrants defined by reference thresholds (Pa = 0.85, confidence = 0.30) categorize fatty acids into distinct therapeutic profiles: high-potential (upper-right), moderate-potential (mixed quadrants), and low-potential (lower-left). Each bubble represents an individual fatty acid candidate.

Systematic pharmacokinetic, safety, and biological activity profiling employed integrated computational platforms (pkCSM, Deep-pk, SwissADME, SOMP, SMP, ADVERPred, PassOnline, PassTarget, KinScreen) across nine fatty acids, revealing distinct structure-property–activity relationships (Table S18, Figure 10, Figure 11 and Figures S6–S8).

Pharmacokinetic profiles demonstrated excellent intestinal absorption (>91% for all compounds), with α-linolenic (ALA), arachidonic (ARA), and linoleic (LA) achieving the highest values (92.2–92.7%). Water solubility varied significantly: linoleic optimal (−4.03 log mol/L) versus ALA/ARA poor (−6.0 log mol/L). Structure-property patterns revealed that ω-6 fatty acids (LA, ARA) occupy the high clearance/hepatotoxicity quadrant, while saturated and ω-3 fatty acids demonstrate superior solubility-clearance combinations despite formulation limitations. Blood–brain barrier permeability showed structural class dependence: oleic/palmitoleic optimal (LogBB −0.327), saturated intermediate (−0.345), ω-6 moderate restriction (−0.619), ALA poorest CNS access (−0.736) (Figure S6). Metabolic vulnerability analysis (SOMP) revealed double bond positions as primary CYP450 sites: ARA highest liability (4 sites, P > 0.9), LA moderate (2 sites), ALA three positions, oleic lowest (1 site). Total clearance predictions showed marked platform discrepancies, with ARA highest (2.10 log mL/min/kg) and myristic lowest (1.69). Pharmacokinetic trade-off analysis (Figure 10) highlighted inverse solubility–clearance relationships positioning compounds into formulation-challenging versus optimal-balanced zones.

Safety profiling established hierarchical toxicity patterns (Figure S6): α-linolenic (ALA) demonstrated the lowest nephrotoxicity risk (Pa = 0.252) and no hepatotoxicity, ω-6 fatty acids (LA, ARA) showed moderate nephrotoxicity (Pa = 0.304) with positive hepatotoxicity signals, while saturated fatty acids ≥C14 displayed dual organ toxicity (nephro-/hepatotoxicity Pa > 0.47) despite optimal metabolic stability. All compounds maintained a bioavailability score of 0.85 despite Lipinski violations (LogP > 5.0).

Biological activity profiling (PassOnline, PassTarget, KinScreen) revealed 147 high-confidence activities (Pa ≥ 0.90) with universal targets including acylcarnitine hydrolase inhibition (Pa = 0.950–0.973) and CYP2J 2 substrate activity (Pa = 0.961–0.974) (Figure S7). Anti-inflammatory selectivity showed prostaglandin-E2 9-reductase inhibition strongest in monounsaturated (Pa = 0.924) versus saturated (Pa = 0.841). PassTarget analysis identified 156 molecular targets (confidence ≥ 0.1) with 23 high-confidence (≥0.3): oxoeicosanoid receptor 1 (0.460–0.469), prostanoid IP receptor (0.391–0.428), fatty acid binding protein (0.358–0.425) (Figure S8). KinScreen demonstrated differential kinase engagement (76 kinases): MAPKAPK5 primary target (oleic/palmitoleic 0.63 > LA/ARA 0.57 > ALA 0.50), PKCα/δ consistent (0.42–0.51), ERK1 variable (0.39–0.54) (Figure 11). Multi-platform integration (Figure 11) positioned ALA in the high-potential quadrant with moderate anti-inflammatory activity (composite Pa = 0.872), superior ACE2/inflammation target confidence (0.335), and balanced kinase engagement (0.42), establishing it as a lead candidate for dual-action therapeutic development.

## 3. Discussion

This computational investigation establishes that naturally occurring fatty acids position as allosteric ACE2 modulators through structure-dependent binding mechanisms [32] with profound therapeutic implications [46] for pandemic preparedness [5]. The computational identification of fatty acid binding sites spatially distinct from ACE2′s catalytic triad (H374, H378, E402) [39] and Spike binding interface (K31–R357) [40] addresses a critical physiological constraint in coronavirus targeting. Quantitative structural analysis demonstrates that predicted binding regions (Regions 1, 3, 5, 7) maintain >15 Å separation from functionally essential domains [35], positioning fatty acids as allosteric modulators rather than competitive inhibitors [37,38]—a mechanistic distinction with profound consequences for therapeutic function.

Conventional orthosteric ACE2 inhibition strategies exacerbate the angiotensin II accumulation already induced by SARS-CoV-2 infection through receptor internalization [21,22,23,24,25], driving cardiovascular complications, pulmonary pathology, and thrombotic cascades characteristic of severe COVID-19 [26,27,28,29]. Our allosteric framework circumvents this pathophysiological constraint by theoretically enabling viral entry disruption without impairing the angiotensin II → angiotensin-(1–7) conversion essential for cardiovascular homeostasis [15,17]. This therapeutic paradigm aligns with emerging experimental evidence demonstrating that allosteric ACE2 binders can inhibit SARS-CoV-2 cellular entry while preserving enzymatic activity, validating the conceptual foundation underlying computational predictions [33,47,48].

Computational dynamics analysis provides a mechanistic rationale for preserved enzymatic function predictions through spatially selective modulation patterns. While α-linolenic acid induced significant conformational stabilization at binding sites (ΔRMSF = −0.280 ± 0.234 Å, Cohen’s d = −1.193, *p* < 10^−10^, Table S16), the catalytic domain (residues 335–430) maintained baseline flexibility (ΔRMSF = +0.024 ± 0.067 Å, *p* = 0.342), suggesting preserved conformational dynamics required for substrate processing [32,48,49,50,51]. Van der Waals-dominated binding (65–80% energetic contribution, Table 1) combined with transient hydrogen bond networks (mean occupancy 64.3 ± 13.9%, Table S15) indicates dynamic conformational modulation rather than rigid blockade. Experimental hydrogen-deuterium exchange mass spectrometry confirms that ligand binding propagates conformational changes >27 Å to modulate ACE2 function [52], establishing precedent for the long-range allosteric effects predicted computationally [53], while maintaining active site accessibility [35].

Computational binding site identification across eight ACE2 regions revealed four primary fatty acid binding hotspots—Regions 1, 3, 5, and 7—spatially distinct from both the catalytic triad [39] and Spike RBD interface [40], as validated by crystallographic quality structure (MolProbity score 1.27, 98.15% Ramachandran-favored residues, Figure S1) [20]. These computationally identified sites diverge mechanistically from experimental allosteric ACE2 modulators: MLN-4760 and DX600 occupy catalytic cleft exosites (~8 Å from zinc center) [54], modulating enzymatic kinetics through local loop restructuring [37], whereas fatty acid binding sites coincide with glycan-free surface patches (>15 Å from catalytic machinery) implicated in Spike allosteric regulation networks [55,56] yet unexploited by small-molecule inhibitors.

Region 3 validation through independent computational screening [57,58] corroborates linoleic acid’s affinity, establishing this region as a druggable allosteric pocket [59] remote from orthosteric sites [60]. The spatial segregation—Regions 1 and 3 maintain >12 Å lateral displacement from principal Spike contact clusters, while Region 7 resides on the C-terminal lobe >20 Å opposite the RBD [35]—positions fatty acids as novel allosteric scaffolds exploiting previously uncharacterized ACE2 surface topology [57].

Evolutionary conservation analysis across mammalian ACE2 orthologs reveals >85% sequence identity within predicted binding regions [61], establishing broad-spectrum therapeutic potential against zoonotic coronavirus emergence that exploits conserved ACE2 recognition machinery. Experimental mutagenesis studies provide functional validation for allosteric coupling mechanisms: perturbations in region one residues reduce Spike affinity ~25% without compromising angiotensin II hydrolysis [38,62], while specific ACE2 mutations modulate viral engagement independently of enzymatic function, directly demonstrating the separation of viral entry from catalytic activity that spatial predictions indicate [38,62]. Molecular interaction profiling reveals that fatty acid binding engages conserved structural features: salt bridge formation with charged residues (LYS74, LYS441, ARG518) provides electrostatic anchoring, while hydrophobic hub residues (LEU95, PHE390, ARG393) create nonpolar complementarity [63,64] accounting for van der Waals dominance (65–80% energetic contribution, Table S17). This dual interaction network–combining directional polar anchors with distributed hydrophobic contacts—enables dynamic conformational modulation propagating distal effects to RBD-contacting helices through interdomain salt bridge pathways [53,65], mechanistically diverging from rigid competitive blockade [66].

MM/PBSA binding free energies (ΔG = −17.45 to −24.12 kcal/mol, Figure 8) require systematic correction for characteristic computational overestimation relative to experimental benchmarks [43,67]. Comparative analysis with established ACE2 inhibitors demonstrates consistent ~8–12 kcal/mol offset: experimental values for MLN-4760 (Ki = 0.34 nM, ΔGexp = −12.3 kcal/mol) and DX600 (Ki = 2.8 nM, ΔGexp = −11.8 kcal/mol) indicate systematic MM/PBSA overestimation observed across multiple computational studies reporting −18.3 to −26.7 kcal/mol ranges [68]. Applying this correction, predicted fatty acid experimental affinities converge to a 1–50 μM range—values consistent with fatty acid-protein interaction precedents (KD = 0.8–2.3 μM for fatty acid binding proteins) [69,70] and validated by allosteric ACE2 binders achieving viral inhibition at IC50 = 14–25 μM without enzymatic impairment [33].

The computational binding hierarchy (oleic: ΔG = −24.12 > arachidonic: −22.34 > α-linolenic: −20.78 > linoleic: −17.45 kcal/mol, Figure 8) directly correlates with van der Waals dominance (65–80% of total binding energy, Table S17), establishing hydrophobic complementarity as the primary structural determinant distinguishing these surface-exposed allosteric binders from electrostatic-dependent active-site inhibitors [64]. Oleic acid’s position as lead candidate emerges from convergent thermodynamic (highest predicted affinity ΔG = −24.12 kcal/mol), kinetic (Fast dynamics τ = 26.4 ns, Neff = 19), and structural (unique flexibilizing effects ΔRMSF = +0.155 Å enabling maintained ACE2 plasticity) criteria.

Multi-platform ADMET profiling (Table S18, Figure S6 hierarchical clustering) established structure-dependent pharmacokinetic and safety profiles positioning ω-3 fatty acids as prioritized translational candidates. Critically, parallel multi-platform biological activity profiling (PassOnline, PassTarget, KinScreen) represents independent computational predictions distinct from ACE2 binding calculations, demonstrating that anti-inflammatory effects derive from structure-based activity recognition against >250,000 reference compounds rather than secondary consequences of ACE2 modulation. All nine fatty acids demonstrated computationally predicted excellent intestinal absorption (>91%), with α-linolenic acid, arachidonic acid, and linoleic acid achieving optimal values (92.2–92.7%, Table S15) exploiting endogenous carrier systems (CD36, FATP1-6, I-FABP) [71,72,73] enabling lymphatic transport bypassing first-pass hepatic metabolism [74,75,76] advantages contrasting sharply with synthetic antivirals requiring intravenous administration (remdesivir oral bioavailability < 1%) [77] or hepatic CYP3A4 inhibition (nirmatrelvir/ritonavir) [78].

Critical safety stratification emerged: ω-3 fatty acids (α-linolenic) exhibited favorable nephrotoxicity profiles (Pa = 0.252, Table S15 green stratification), contrasting with remdesivirs’s documented acute kidney injury incidence (5–10%) [79,80,81], while hepatotoxicity predictions flagged concerning signals for ω-6 fatty acids requiring systematic validation through hepatocyte cytotoxicity assays [82,83,84]. This computational stratification establishes α-linolenic acid as a priority lead candidate integrating an optimal safety profile, high absorption (92.2%), and pronounced protein stabilization capacity (ΔRMSF = −0.280 Å, Table S16).

Primary translational challenge involves aqueous solubility limitations (log S < −4.0 polyunsaturated compounds, Table S18) addressable through established lipid-based delivery systems. Self-nanoemulsifying drug delivery systems (SNEDDS) incorporating medium-chain triglycerides and Tween 80 surfactant have demonstrated 8–10-fold bioavailability enhancement for ω-3 fatty acids [85], achieving <100 nm droplet sizes with rapid gastrointestinal dispersion (<10 min) [86] and establishing regulatory precedent through marketed products (Omacor^®^, Lovaza^®^) [87]. For linoleic/arachidonic acids, hepatotoxicity signals necessitate lymphatic-targeting formulations enabling intestinal lymphatic absorption, bypassing portal circulation, reducing liver exposure 60–75% while maintaining bioavailability [75,88]—alternatively, inhalational delivery routes enable direct pulmonary ACE2 targeting, achieving lung:plasma ratios > 100:1, circumventing systemic hepatic exposure [89]. Critical limitations requiring experimental validation, Albumin binding dynamics, and free fatty acid bioavailability under physiological conditions remain computationally predicted rather than experimentally confirmed. Phase 3 pseudovirus neutralization assays with lipoprotein-containing serum and Phase 4 in vivo pharmacokinetic studies will definitively establish whether computationally predicted ACE2 accessibility translates to therapeutically relevant modulation despite plasma protein binding (~99% albumin association for circulating fatty acids).

Translation of computational predictions into therapeutically validated candidates requires phased experimental validation encompassing, (1) binding thermodynamics (SPR, ITC) establishing KD ≤ 50 μM with spike-competitive inhibition [33,90], (2) preserved enzymatic function (≥70% retained Vmax confirming allosteric mechanism) [91,92], (3) pseudovirus neutralization (IC50 with selectivity index > 10) [33,93,94], (4) cardiovascular safety in vivo confirming RAAS homeostasis [95,96], and (5) lipid-based formulation achieving oral bioavailability >20% [85,86,97]. This systematic pathway (timeline: 18–30 months) establishes proof-of-concept for computational to experimental translation applicable to future pandemic preparedness applications [5].

Host-directed fatty acid modulators targeting conserved ACE2 receptor architecture (>85% mammalian ortholog sequence conservation) [61] provide broad-spectrum pandemic preparedness potential, circumventing variant escape vulnerabilities inherent to RBD-specific therapeutics. Omicron lineages demonstrated 15–30-fold reduced neutralization by monoclonal antibodies [98] effective against ancestral strains [99], exposing critical limitations of variant-specific approaches [9,10]. Host receptor targeting exploits evolutionary constraints fundamental to viral tropism; Coronaviruses require ACE2 binding for cellular entry across phylogenetically diverse strains [18], rendering therapeutic strategies targeting conserved host machinery resistant to viral mutation-driven escape (10^−6^–10^−4^ substitutions/site/cycle RNA virus mutation rates) [11].

Comparative analysis with established ACE2-targeting strategies reveals distinct mechanistic trade-offs positioning fatty acids within differentiated therapeutic space. Competitive active-site inhibitors (MLN-4760, DX600) achieve nanomolar potency (Ki = 0.34–2.8 nM) [54] but fundamentally compromise angiotensin II degradation [37,100], while monoclonal antibody approaches demonstrate picomolar binding yet face variant escape (Omicron 15–30-fold reduced neutralization) [98,99], prohibitive cost ($2500–5000/dose) [101], intravenous requirements limiting prophylaxis, and antibody-dependent enhancement risks [102]. Fatty acids offer counterpoint advantages, host-directed mechanism resistant to viral evolution [9,61], predicted oral bioavailability (>91% intestinal absorption exploiting endogenous carriers, Table S18) [71,72,73,74], and cost-effectiveness reflecting natural product sourcing [103,104], establishing reproducible workflows for emergency response applications against future zoonotic spillovers exploiting ACE2 homology across mammalian hosts [1,2].

The computational framework establishes a methodology fully generalizable to fatty acids from any source—plant oils, marine oils, synthetic production, or natural lipid-rich organisms. Systematic evaluation demonstrates that binding selectivity emerges from optimized structure–function relationships rather than source-dependent constraints alone.

However, the computational prediction that distinct fatty acid classes exhibit complementary binding kinetics represents a hypothesis requiring experimental validation. Oleic acid’s Fast dynamics (τ = 26.4 ns, Neff = 19) suggest rapid conformational modulation suitable for acute intervention, while α-linolenic acid’s slower kinetics (τ = 303 ns, though limited sampling Neff = 2) predict sustained stabilization—mechanistically divergent roles within predicted “balanced” multi-agent combinations. Whether such compositional balancing yields synergistic antiviral efficacy (i.e., combination IC50 < single-agent IC50 values) remains unvalidated and represents a critical Phase 2 research objective through pseudovirus neutralization assays comparing (a) oleic acid monotherapy, (b) α-linolenic acid monotherapy, and (c) balanced oleic + α-linolenic combinations.

Notably, gas chromatography-mass spectrometry analysis of certain insect-derived lipid-rich sources demonstrates balanced multi-class distribution [105], oleic acid (28–40%), linoleic acid (12–20%), α-linolenic acid (2–5%), and arachidonic acid (1–3%) [103,104,105,106,107,108]—a compositional profile substantially different from conventional agricultural sources exhibiting class-specific monopolization. No single conventional agricultural source naturally provides equivalent balanced multi-class distribution, olive oil exhibits ω-9 monopoly (70–80% oleic, <1% ω-3) [109], flaxseed oil demonstrates ω-3 excess (50–60% α-linolenic, oxidation-prone) [110], fish oil concentrates marine ω-3 with sustainability concerns [111], and sunflower/soybean oils show ω-6 dominance (65–75% linoleic) [112,113]. Establishing superior natural sources for therapeutic deployment; therefore, requires parallel tracks: (1) Direct Phase 1–4 validation of existing multi-class natural sources exhibiting the predicted compositional profile, and (2) Agricultural/biotechnological development of optimized lipid-rich sources engineered toward the computationally predicted balanced compositions.

This computational investigation establishes that naturally occurring fatty acids represent promising allosteric ACE2 modulators for systematic experimental validation within host-directed pandemic preparedness frameworks. Additionally, this study establishes fatty acid relative ranking efficacy for experimental prioritization rather than claiming absolute affinity prediction accuracy. Experimental validation via Surface Plasmon Resonance (SPR) and Isothermal Titration Calorimetry (ITC) represents the critical next step for absolute binding thermodynamic quantification. Oleic acid emerges as immediate lead candidate integrating optimal predicted affinity (ΔG = −24.12 kcal/mol), Fast dynamics enabling rapid conformational modulation, and unique flexibilizing effects maintaining ACE2 physiological function, while α-linolenic acid prioritization for translational development combines a favorable safety profile (nephrotoxicity Pa = 0.252), pronounced protein stabilization (ΔRMSF = −0.280 Å), and oral delivery feasibility. Phased experimental validation (timeline: 18–30 months) will directly test computational predictions, establishing proof-of-concept for computational to experimental pipelines addressing future pandemic threats through conserved host receptor targeting resistant to viral evolutionary escape.

## 4. Materials and Methods

We systematically evaluated nine fatty acids binding to eight ACE2 regions through an integrated computational workflow. SwissDock docking identified binding sites; 100 ns GROMACS/CHARMM36m simulations characterized dynamics; MM/PBSA calculations estimated binding free energies; linear discriminant analysis extracted mechanistic patterns. This approach screened 72 combinations with 20–50× greater computational efficiency than alchemical free energy methods, establishing reproducible protocols for natural product evaluation in pandemic contexts.

### 4.1. Compound Selection and Preparation

Nine fatty acids representing >85% of total lipid profile were selected, oleic acid (CID 445639), arachidonic acid (CID 444899), palmitic acid (CID 985), stearic acid (CID 5281), myristic acid (CID 11005), linoleic acid (CID 5280450), palmitoleic acid (CID 445638), alpha-linolenic acid (CID 5280934), and margaric acid (CID 10465) [103,104,105,106,107,108]. Three-dimensional structures were retrieved from the PubChem database [114] and energy-minimized using Avogadro v1.98.0 [115] with the MMFF94s force field [116] and the steepest descent algorithm until convergence (dE = 0.0 kcal/mol·Å). Optimized structures were converted to mol2 format using Open Babel v3.1.1 [117] for docking compatibility (Figure S9). All computational data, molecular dynamics trajectories, ADMET predictions, and methodological protocols are available (Zenodo: 10.5281/zenodo.16846914, CC BY 4.0 License).

### 4.2. Protein Selection and Preparation

Human ACE2 complexed with SARS-CoV-2 Spike receptor binding domain (PDB: 6M0J, 2.45 Å resolution) [20] was prepared using UCSF ChimeraX v1.8 [118] and WinCoot v0.9.8.95 [119]. Water molecules and existing ligands were removed, hydrogen atoms added, and geometry optimized using standard crystallographic refinement protocols to resolve steric clashes. Structure validation via MolProbity v4.5.2 [120] evaluated stereochemical parameters and atomic clashes (Figure S1). AutoDockTools v1.5.7 [121] prepared docking parameters (grid generation, atomic charge assignment) (Figure S9). Eight ACE2 binding regions were identified through integrated computational and the literature-based analysis [122,123]. DoGSiteScorer [124,125] predicted druggable pockets based on geometric properties (volume, depth, hydrophobicity). The literature cross-referenced Reactome (functional domains) [126], BindingDB (inhibitor sites) [127], UniProt (catalytic residues H374/H378/E402; Spike interface K31–R357) [128], and crystallographic data [20] (Figure S9). Regions 1, 3, 5, 7: allosteric sites (15–18 Å from catalytic/Spike interfaces) enabling selective modulation. Regions 2, 4, 6, 8: peripheral regulatory sites.

The eight computationally defined binding regions represent grid-box search spaces strategically positioned to enable allosteric modulation rather than orthosteric inhibition. Regions 1, 3, 5, and 7 (classified as primary allosteric sites) maintain >15 Å spatial separation from both the catalytic triad (H374/H378/E402, residues 335–430) responsible for angiotensin II processing and the Spike-binding interface (K31–R357, residues 19–85) mediating viral entry, theoretically enabling selective disruption of viral attachment without impairing enzymatic function. Region 1 (residues 30–110, N-terminal domain) coincides with the Spike recognition periphery, exhibiting hydrophobic patches (PHE40, LEU73, PHE390, LEU391) and bifunctional anchoring residues (LYS74) enabling fatty acid binding through combined van der Waals and electrostatic interactions while positioned >12 Å lateral to principal Spike contact clusters. Region 3 (residues 150–280, interdomain linker) occupies a glycan-free surface cleft between the N-terminal peptidase and C-terminal collectrin-like domains, validated through independent computational screening [57,58] as a druggable allosteric pocket remote from orthosteric sites. Region 5 (residues 280–450, catalytic domain periphery) resides along the α-helical scaffold flanking but not overlapping the active site, with key hydrophobic residues (ILE291, ALA413, PHE438, ILE446) and electrostatic anchors (ARG518, LYS441) creating binding pockets capable of propagating conformational effects to the catalytic machinery through interdomain communication pathways while preserving direct substrate access. Region 7 (residues 450–580, C-terminal collectrin-like domain) is positioned on the opposite face of ACE2 relative to the Spike RBD (>20 Å displacement), representing a mechanistically distinct allosteric site implicated in homodimer formation and membrane anchoring.

Regions 2, 4, 6, and 8 (peripheral regulatory sites) occupy glycosylation-adjacent patches and interdomain hinge regions implicated in conformational regulation but exhibiting lower druggability scores and reduced fatty acid binding affinity in preliminary docking (mean SP-ΔG = −6.45 ± 0.31 kcal/mol vs. −6.92 ± 0.22 kcal/mol for primary allosteric sites, Table S1). This spatial architecture positions fatty acid binding sites within allosteric communication networks capable of propagating conformational effects to functionally critical domains while preserving catalytic machinery integrity and Spike recognition surface—establishing the mechanistic foundation for dual viral entry disruption and RAAS homeostasis preservation through selective allosteric modulation rather than complete receptor blockade.

### 4.3. Molecular Docking Protocol

Flexible molecular docking was performed using the SwissDock web server [129] with EADock DSS engine and CHARMM force field [130], ensuring parameter consistency with subsequent MD simulations. Protocol validation employed redocking of co-crystallized N-acetyl-D-glucosamine (NAG) to its original binding site (ASN90) using a 22 Å^3^ cubic grid box. For each fatty acid, 50 poses were generated using ‘Accurate’ mode with medium sampling exhaustivity [131,132]. Full ligand flexibility is enabled alongside side-chain flexibility for 23 critical ACE2 residues (HIS34, GLU37, ASP38, TYR41, GLN42, LYS74, MET82, TYR83, ASN90, LYS94, PHE274, GLU329, LYS353, ASP355, ARG357, ARG393, GLU406, LYS441, TYR449, THR445, HIS505, TYR515, ARG518; Figure S10). Poses were ranked by SwissDock SP–ΔG (empirical ΔG_bind_ weighted sum of van der Waals, electrostatic, and solvation terms) and FullFitness scores, with selection thresholds SP–ΔG < −6.00 kcal/mol and FullFitness < −5.00.

This approach provided optimal computational efficiency (~36 CPU-hours for 72 systems) [129,133] compared to alchemical methods FEP/TI (~144,000–360,000 CPU-hours, 4000–10,000× increase) [134,135], enabling systematic screening while maintaining relative binding affinity ranking accuracy for structurally related fatty acids [136]. Top-ranked poses were analyzed using LigandScout 4.5 (pharmacophore mapping) [137], BIOVIA Discovery Studio Visualizer 2024 (3D interaction networks) [138], Maestro Schrödinger 2024-1 (binding mode visualization) [139], and PLIP web tool (interaction profiling) [140]. Final candidates selected for MD simulations, alpha-linolenic (Regions 1, 2, 5), arachidonic (Regions 1, 2, 7), linoleic (Regions 1, 3, 5), oleic (Regions 1, 5, 7).

Statistical analyses in R v4.2.0 [141] included Shapiro–Wilk [142] normality testing (*p* > 0.30), Levene’s variance homogeneity assessment (*p* = 0.45) [143], and independent two-tailed *t*-test comparing unsaturated versus saturated fatty acids (t_7_ = −1.69, *p* = 0.135, mean difference −0.16 kcal/mol, 95% CI [−0.36, +0.04], Cohen’s d = −1.13, post hoc power = 52%) [144].

### 4.4. Molecular Dynamics Simulations Protocol

MD simulations were performed using GROMACS v2023.4 [145] with CHARMM36m force field (July 2017 version) [146,147], selected for (1) validated performance in protein-lipid simulations with established fatty acid parameters [148,149], (2) consistency with SwissDock CHARMM protocol (Section 4.3) ensuring seamless docking-to-MD transition, and (3) the literature precedent for ACE2-ligand studies [150]. Force field limitations, fixed-charge approximation without electronic polarization limit accuracy for cation-π interactions (LYS74, LYS441, ARG518) [151], systematic salt-bridge over-stabilization (0.8–2.9 kcal/mol versus polarizable fields) [152,153], and polyunsaturated fatty acid parametric uncertainty from limited ω-3/ω-6 protein-binding training data [154]. Polarizable force fields (CHARMM Drude, AMOEBA) [155,156] provide superior accuracy for charge transfer and polarization phenomena [157] but require 2–4× computational cost [155], prohibiting systematic application across 72 initial screening combinations.

System preparation utilized CHARMM-GUI [147] with cubic boxes, TIP3P solvation [158], 0.15 M NaCl, and periodic boundary conditions. Steepest descent minimization (maximum force < 1000.0 kJ/(mol·nm)) [145] employed harmonic restraints (backbone 400.0 kJ/(mol·nm^2^), side chains 40.0 kJ/(mol·nm^2^)) [159] preserving crystallographic structure. Two-stage equilibration, (1) NVT (100 ps, 303.15 K, V-rescale τ = 0.1 ps) [160] for temperature stabilization, (2) NPT (1 ns, 303.15 K, 1 bar, Parrinello–Rahman τ = 2.0 ps, compressibility 4.5 × 10^−5^ bar^−1^) [161] for density/pressure equilibration. Production simulations (100 ns, 2 fs timestep, NPT): V-rescale (303.15 K, τ = 0.1 ps), Parrinello–Rahman (1 bar, τ = 2.0 ps), Verlet cutoff (1.2 nm) [162], force-switch van der Waals (1.0–1.2 nm) [163], PME electrostatics (1.2 nm cutoff, 0.16 nm grid, 4th order) [164], LINCS constraints (order 4) [165], center-of-mass removal (every 100 steps) [166], trajectory storage (10 ps intervals).

The 100 ns duration was selected based on, (1) convergence analysis demonstrating 75% systems (Fast/Intermediate dynamics) achieved statistical convergence (Neff = 4–19, variance < 0.1), (2) the literature precedent establishing 100 ns as sufficient for allosteric protein–small molecule binding in comparable systems (ACE2: 805 residues) [150,167], capturing local binding pocket rearrangements (10–100 ns experimental timescales) as demonstrated for fatty acid binding proteins [168,169] and ACE2 inhibitor complexes, and (3) computational feasibility enabling systematic 72-combination screening. Acknowledged limitations, slow-dynamics systems (τ = 190–303 ns, 25%, Neff = 2–3) require extended simulations (200–500 ns); complete binding/unbinding cycles (ms–s), extensive domain reorganization (μs), and long-range allosteric communication (>500 ns) [170,171] necessitate enhanced sampling (replica exchange MD [172], metadynamics [173]).

Multi-parameter structural analysis employed five orthogonal metrics: (1) backbone (Cα) RMSD for global fold preservation, (2) ligand RMSD for binding pose consistency, (3) per-residue RMSF for flexibility, (4) SASA for surface accessibility, and (5) radius of gyration for compactness. Backbone RMSD was prioritized because (1) it reflects global conformational stability filtering side-chain rotameric noise, (2) it is established in the literature precedent as a primary convergence metric, and (3) its side-chain flexibility is independently quantified via RMSF. RMSD values of 2.5–3.5 Å fall within expected ranges for fatty acid–protein complexes due to inherent ACE2 flexibility (805-residue membrane enzyme with conformational dynamics) [150,167], fatty acid–induced flexibility (C16–C20 chains), and allosteric site characteristics (flexible loops/hinges). The literature validates comparable systems: 2.5–4.0 Å (fatty acid binding proteins) [168], 2.0–3.5 Å (membrane enzymes with lipid ligands), 2.5–4.5 Å (flexible domains during allosteric transitions) [170], as reported for analogous membrane-associated enzyme systems. Quality thresholds: backbone RMSD > 3.5 Å or ligand RMSD > 15.0 Å flagged; all simulations demonstrated stable structures with temperature fluctuations < ±5 K, pressure fluctuations within ±50 bar, and total energy drift <0.5% over 100 ns.

Equilibration detection: sliding-window Mann–Whitney U tests (window = 50 frames, *p* > 0.05), validated via Kolmogorov–Smirnov tests (α = 0.05). Convergence: block averaging (block size = max (50, N/10)), variance ratios < 0.1, integrated autocorrelation times (τint) with statistical inefficiency g = 2τint + 1 and Neff = N/g. Statistical validation: linear drift tests, Shapiro–Wilk normality, stationarity (Augmented Dickey–Fuller/KPSS), LOWESS smoothing (frac = 0.1), 95% confidence intervals (sliding window = 50 frames), bootstrap resampling (10,000 iterations), Benjamini–Hochberg FDR correction (α = 0.05), Cohen’s d effect sizes.

### 4.5. Binding Free Energy Calculations

MM/PBSA calculations were performed using gmx_MMPBSA v1.5.2 to estimate relative binding free energies (ΔG_bind_) for all fatty acid–ACE2 complexes. Computational efficiency requirements necessitated MM/PBSA instead of alchemical methods, MM/PBSA (~100 CPU-hours per system × 12 = 1200 h total) versus FEP/TI (~2000–5000 CPU-hours per system × 12 = 24,000–60,000 h, 20–50× increase), rendering FEP/TI prohibitive for systematic 72-combination initial screening. MM/PBSA excels in relative binding affinity ranking (R^2^ = 0.6–0.8 for structurally related compounds) with characteristic systematic overestimation (~8–12 kcal/mol versus experimental) [174] due to entropic approximations and implicit solvation limitations. The literature validation: MM/PBSA predictions (ΔG = −17.45 to −24.12 kcal/mol) align with published fatty acid-protein studies; comparison with experimental ACE2 inhibitors (MLN-4760: Ki = 0.34 nM, ΔG_exp_ ≈ −12.3 kcal/mol; DX600: Ki = 2.8 nM, ΔG_exp_ ≈ −11.8 kcal/mol) [53] confirms methodological consistency for relative ranking.

Sampling strategy: 1000 snapshots from equilibrated trajectories (final 80 ns, every 80 ps) ensured statistical representation. Energy decomposition: ΔG_bind_ = ΔE_MM + ΔG_solv_ (entropic contributions omitted, ~1000× computational cost). Solvation energy: polar component via Generalized Born (igb = 5 variant, mbondi2 radii), non-polar from solvent-accessible surface area (γ = 0.0226778 kJ/(mol·Å^2^), b = 3.84928 kJ/mol). Dielectric environment: ε_solute_ = 1, ε_solvent_ = 80, ionic strength 0.150 M. Statistical analysis: standard deviations from 1000 snapshots, bootstrapping (100 resamples) for 95% confidence intervals, per-residue decomposition identifying key binding residues, block averaging (block size = 50 frames, variance ratios < 0.1) assessing convergence.

Acknowledged limitations: omitted entropy (±4–6 kcal/mol uncertainty), implicit solvation cannot capture explicit water-mediated interactions, single-trajectory protocol assumes identical conformational sampling, force field dependence (CHARMM36m parameter uncertainty for polyunsaturated fatty acids 15–25%). Experimental validation via Surface Plasmon Resonance (SPR), Isothermal Titration Calorimetry (ITC), and competitive binding assays represents the next critical step for absolute affinity quantification and therapeutic development confirmation.

### 4.6. Linear Discriminant Analysis for Energy Component Discrimination

To complement unsupervised PCA, supervised Linear Discriminant Analysis (LDA) [72] maximized energetic separation between ACE2 regions and fatty acid classes, revealing mechanistic patterns masked by global variance. Unlike PCA (unsupervised, maximum variance independent of labels) [44], LDA explicitly optimizes projection axes, minimizing within-class scatter (S_W_) while maximizing between-class scatter (S_B_), extracting region-specific and fatty acid-specific energy signatures. This dual-method strategy (PCA: exploratory variance structures; LDA: mechanistic classification patterns) represents best practices in MD energy landscape analysis, comprehensively characterizing complex binding phenomena through orthogonal dimensionality reduction [175].

Data preprocessing: MM/PBSA energy trajectories from post-equilibration segments (final 80 ns, determined via Mann–Whitney U tests, window = 50 frames, *p* > 0.05) were processed to extract seven energy components (ΔE_vdW_, ΔE_elec_, ΔG_polar_, ΔG_nonpolar_, ΔE_gas_, ΔG_solv_, ΔG_bind_). Stratified random sampling (n = 200 frames per region, seed = 42) yielded balanced datasets (600 samples per fatty acid). Z-score standardization (X_scaled_ = (X − μ)/σ) ensured large-magnitude components (e.g., unfavorable electrostatics + 262 kcal/mol) did not numerically dominate discriminant construction.

LDA applied three complementary frameworks, (1) Fatty acid classification (4 classes → LD1-LD3): discriminates α-linolenic, arachidonic, linoleic, oleic independent of binding site, revealing conserved recognition features; (2) Regional classification (5 regions → LD1-LD4): isolates ACE2 binding site energetic microenvironments determining selectivity; (3) Combined classification (12 combinations → LD1-LD5): identifies synergistic fatty acid-region effects optimizing structure activity relationships. Mathematical formulation: generalized eigenvalue problem SB·w = λ·S_W_·w maximizes Fisher’s criterion J(w) = (w^T^ S_B_ w)/(w^T^ S_W_ w). Singular value decomposition (SVD) ensured numerical stability for collinear components (ΔE_gas_ = ΔE_vdW_ + ΔE_elec_).

Clustering quality: Silhouette coefficient, Calinski–Harabasz index, and Davies–Bouldin index quantified discrimination performance with statistical significance (chi-square tests, α = 0.001) compared to random baselines (25%, 20%, 8.3%, respectively). Implementation: scikit-learn v1.3.0 (LinearDiscriminantAnalysis, solver = ‘svd’), Python 3.10. Complete workflows are provided in Supplementary Code S1, ensuring reproducibility. Per-residue decomposition identified key energy component drivers of regional/fatty acid discrimination, with discriminant coefficients quantifying each MM/PBSA component contribution to mechanistic separation. Complete LDA workflows, including preprocessing, equilibration detection, stratified sampling, standardization, and multi-level classification, are available in the Zenodo repository (DOI: 10.5281/zenodo.16846914), ensuring complete transparency and reproducibility.

### 4.7. ADMET Analysis and Biological Activity Predictions

Biological activity screening via PASS Online [176,177] identified pharmacological potential through structure–activity relationship (SAR) analysis of >250,000 reference compounds predicting >3500 biological activities. High-confidence predictions (Pa > 0.70) targeted three therapeutic areas: renin-angiotensin system regulation (ACE2 modulation, angiotensin processing), direct viral target interactions (antiviral mechanisms, viral protein inhibition), and immunomodulation (cytokine regulation, inflammatory enzyme inhibition (Figure S11).

ADMET profiling employs a consensus approach integrating three platforms: pkCSM (https://biosig.lab.uq.edu.au/pkcsm/, accessed on 18 September 2024) (graph-based molecular signatures, machine learning on pharmaceutical datasets) [178], Deep-PK (https://biosig.lab.uq.edu.au/deeppk/, accessed on 19 September 2024) (physiologically based pharmacokinetic modeling) [179], and SwissADME (https://www.swissadme.ch/, accessed on 20 September 2024) (rule-based drug-likeness: Lipinski’s Rule of Five, Veber’s criteria) [180] (Figure S11). Integrated assessment encompassed absorption (intestinal, Caco-2, skin permeability), distribution (volume of distribution, plasma protein binding, blood–brain barrier), metabolism (CYP450 profiles: CYP1A2, 2C9, 2C19, 2D6, 3A4), excretion (renal clearance, P-glycoprotein status), toxicity (AMES, hERG, hepatotoxicity, nephrotoxicity, skin sensitization).

Metabolic fate via Way2Drug platforms: SOMP (Phase I/II metabolic sites, ΔP > 0.5) [181], SMP (CYP isoform/UGT specificity, Pa > 0.5) [182]. Protein target prediction: KinScreen (kinase targets, immunomodulatory signaling) [176,177], PASS Target (secondary pharmacological targets) [183]. All predictions referenced Reactome, KEGG, and STRING databases for mechanistic validation. Quality control: platform-specific default parameters, consensus scoring from ≥2 independent methods, confidence scores documented for experimental prioritization.

## 5. Conclusions

Natural fatty acids emerge as the first systematically evaluated allosteric ACE2 modulators identified through integrated computational screening. Lead candidate, oleic acid (ΔG = −24.12 kcal/mol), demonstrates computationally predicted favorable binding within the ensemble while maintaining critical spatial segregation (>15 Å) from both the ACE2 catalytic triad (H374–H378–E402) and the SARS-CoV-2 Spike binding interface (K31–R357), establishing relative ranking for experimental validation. This spatial architecture uniquely enables allosteric modulation distinct from conventional competitive inhibition.

Unlike conventional ACE2 antagonists (MLN-4760 Ki = 0.34 nM, DX600 Ki = 2.8 nM) that occupy the catalytic site and trigger pathological angiotensin II accumulation, computational predictions indicate that fatty acid binding sites theoretically enable simultaneous viral entry disruption and preservation of essential physiological RAAS functional dual mechanism, which is mechanistically impossible for orthosteric inhibitors. This architecture circumvents the fundamental dilemma that complete ACE2 inhibition exacerbates COVID-19 pathophysiology through angiotensin II accumulation.

Comprehensive binding energy decomposition reveals that fatty acid–ACE2 interactions derive overwhelmingly from hydrophobic complementarity (74.3% of interaction energy) rather than electrostatic interactions characteristic of competitive inhibitors. This hydrophobic-driven mechanism suggests intrinsic resistance to SARS-CoV-2 Spike variant evolution, as mutations enabling viral escape from lipid-based interactions would simultaneously compromise viral fitness through altered membrane anchoring and fusion machinery function.

The integrated framework—molecular docking (36 CPU-hours for 72 systems), 100 ns molecular dynamics (GROMACS/CHARMM36m), MM/PBSA binding energetics, and Linear Discriminant Analysis—achieves 20–50× superior computational efficiency compared to alchemical free energy methods (1200 CPU-hours vs. 24,000–60,000 h for FEP/TI) while maintaining relative ranking accuracy for structurally related compounds (R^2^ = 0.6–0.8). This efficiency establishes reproducible, scalable protocols enabling systematic natural product evaluation for pandemic preparedness—a critical capability as novel respiratory pathogens emerge.

Fatty acids derived from diverse renewable biological sources (plant oils, marine organisms, insect-derived lipids, microbial fermentation, and chemical synthesis) represent economically sustainable alternatives to chemically synthesized therapeutics. This accessibility potentially enables equitable global distribution during pandemic emergencies, addressing a critical limitation of current monoclonal antibody and small-molecule approaches requiring expensive manufacturing infrastructure.

This investigation positions natural fatty acids within a rational computational to experimental drug discovery pipeline. The framework bridges ethnomedicine-derived natural products with cutting-edge molecular analytics, establishing scientific credibility for accelerated development. A phased experimental validation strategy (18–30 months) will definitively establish proof-of-concept through, (1) Phase 1 (Months 1–6): SPR/ITC binding thermodynamics confirmation; (2) Phase 2 (Months 4–12): ACE2 enzymatic activity preservation and conformational characterization; (3) Phase 3 (Months 8–18): Pseudovirus neutralization against ancestral, Delta, and Omicron variants with selectivity indices; (4) Phase 4 (Months 12–30): In vivo safety, pharmacokinetics, and formulation optimization. Success would position natural fatty acids as complementary pandemic preparedness tools within diversified antiviral portfolios.

This computational investigation establishes relative ranking efficacy for experimental prioritization rather than absolute affinity prediction accuracy. Oleic acid emerges as immediate lead candidate for experimental validation integrating highest predicted affinity within the computational hierarchy (ΔG = −24.12 kcal/mol), Fast dynamics enabling rapid conformational modulation, and unique flexibilizing effects theoretically maintaining ACE2 physiological function, while α-linolenic acid prioritization for translational development combines favorable safety profile (nephrotoxicity Pa = 0.252), pronounced protein stabilization (ΔRMSF = −0.280 Å), and oral delivery feasibility. Experimental validation via Surface Plasmon Resonance (SPR) and Isothermal Titration Calorimetry (ITC) represents the critical next step for absolute binding thermodynamic quantification, confirming computational predictions and establishing proof-of-concept for natural product-based pandemic preparedness strategies.

This work demonstrates that systematic computational screening of natural products—informed by structural biology, energetics modeling, and statistical pattern recognition—can identify promising therapeutic candidates from renewable, accessible biological sources within clinically relevant timeframes. By combining allosteric-modulation principles with natural product chemistry, this framework fundamentally reshapes pandemic preparedness strategies, transitioning from single-mechanism synthetic drugs toward multi-target, evolutionarily resistant, sustainable therapeutic platforms.

All conclusions represent computational hypotheses requiring systematic experimental confirmation. The 100 ns simulation timescales, while capturing local binding pocket rearrangements (10–100 ns experimental timescale), provide limited conformational sampling; slow-dynamics systems (25%, τ = 190–303 ns) require extended simulations (200–500 ns). MM/PBSA predictions demonstrate characteristic systematic biases (8–12 kcal/mol overestimation) requiring correction through experimental calibration with SPR and ITC. Entropy contributions (~4–6 kcal/mol) are omitted, and implicit solvation cannot capture explicit water-mediated interactions. Polyunsaturated fatty acid parametric uncertainty (15–25%) necessitates validation. Future computational refinement incorporating enhanced sampling (replica exchange MD, metadynamics) and explicit solvation will improve predictive accuracy. This integrated computational to experimental framework positions natural fatty acids as scientifically credible pandemic preparedness candidates, provided rigorous experimental validation confirms therapeutic potential, and establishes safety and efficacy.

## Figures and Tables

**Table 1:** Molecular Interaction Profiles for Lead Fatty Acids in Key ACE2 Binding Regions.

Fatty Acid	ACE2 Region	SP-dG (kcal/mol)	Key Hydrophobic Contacts	C–H Bonds	H-Bonds	Salt Bridges
**α-Linolenic acid**	1	−6.96	PHE40, LEU73, PHE390, LEU391, ARG393	—	LYS74	LYS74
	2	−6.97	LEU91, THR92, LEU95, VAL209, VAL212, PRO565	ASN90	LEU91	—
	5	−7.01	ILE291, PRO415, GLU435, PHE438, LYS441, HIS540	—	THR445	LYS441
**Arachidonic acid**	1	−7.06	PHE40, LEU73, LEU100, PHE390, LEU391, ARG393	LYS74	LYS74	—
	2	−7.02	LEU91, LEU95, VAL209, VAL212, PRO565	—	TYR202	—
	7	−7.28	LEU91, LEU95, VAL209, VAL212, PRO565	ASN90	LEU91	—
**Linoleic acid**	1	−7.00	LEU73, PHE390, LEU391, ARG393	—	LYS74	LYS74
	3	−7.25	LEU156, LEU266	LYS441	LYS441	—
	5	−6.95	ILE291, ALA413, PHE438, ILE446	—	—	ARG518
**Oleic acid**	1	−7.12	PHE40, LEU73, LEU100, PHE390	LYS74	—	LYS74
	5	−6.97	ILE291, ALA413, PHE438, ILE446	—	—	ARG518
	7	−6.79	ILE291, LEU370, LEU410, ALA413, PHE438	—	THR445	—

Data represent the lowest-energy poses from 50 conformational sampling runs per region. Selection criteria: SP-ΔG ≤ −6.70 kcal/mol and regional coverage ≥37.5% (≥3 of 8 regions). Interaction detection thresholds: hydrophobic contacts ≤ 3.5 Å; C–H bonds ≤ 3.5 Å; hydrogen bonds ≤ 3.5 Å; salt bridges ≤ 4.0 Å. “—” indicates no interactions for the specified pose.

## Data Availability

All data, analysis results, and input files supporting the findings of this study are publicly available in a permanent Zenodo repository at the following digital object identifier (DOI): https://doi.org/10.5281/zenodo.16846914. The deposited data package is organized into seven main folders, each containing a specific README.txt file for detailed explanations. The repository includes: the initial and optimized protein structures; the input files and 3D structures for all nine fatty acids; the complete outputs from ADMET and bioactivity predictions; the summary and raw output files from molecular docking; the complete GROMACS input files (structure, topology, parameters) for all 12 molecular dynamics simulations; the results from the MM/PBSA binding free energy calculations; and the underlying data for all figures and tables presented in the manuscript. All software and web servers used in this study are publicly available and detailed in the Methodology section and the repository’s README files.

## Supplementary Materials

The following supporting information can be downloaded at: https://www.mdpi.com/article/10.3390/ijms27010402/s1.

## Abbreviations

The following abbreviations are used in this manuscript:

ACE2	Angiotensin-Converting Enzyme 2
ARDS	Acute respiratory distress syndrome
RAAS	The renin–angiotensin–aldosterone system
ALA	Alpha-linolenic
ARA	Arachidonic
LA	Linoleic
OA	Oleic
Rg	Radius of Gyration
SASA	Solvent Accessible Surface Area
RMSD	Root-mean-square deviation
RMSF	Root Mean Square Fluctuation
MM/PBSA	Molecular Mechanics/Poisson-Boltzmann Surface Area
