# Supplementary material for: Natural Fatty Acids as Dual ACE2-Inflammatory Modulators: Integrated Computational Framework for Pandemic Preparedness
Source: Int J Mol Sci. 2025 Dec 30;27(1):402. doi: 10.3390/ijms27010402 (PMC12787236; doi:10.3390/ijms27010402)
Supplement: Supplementary file 1 [file ijms-27-00402-s001.zip › ijms-3996292-supplementary.pdf]

Figure S1. Validation of the structural quality of the ACE2 receptor (PDB 6M0J). MolProbity assessment verifies the appropriateness of the structure for simulations. The Ramachandran plot (top center panel) reveals an excellent topology, with 98.15% of residues in preferred areas and 0% in unfavorable areas. The statistics table (lower section) presents outstanding metrics, featuring a MolProbity score of 1.27 and a Clashscore of 3.14, confirming the atomic geometry and the lack of considerable steric hindrance. The green highlights in the table indicate values that meet the recommended quality goals. These findings validate the dependability of the framework for docking and molecular dynamics investigations.

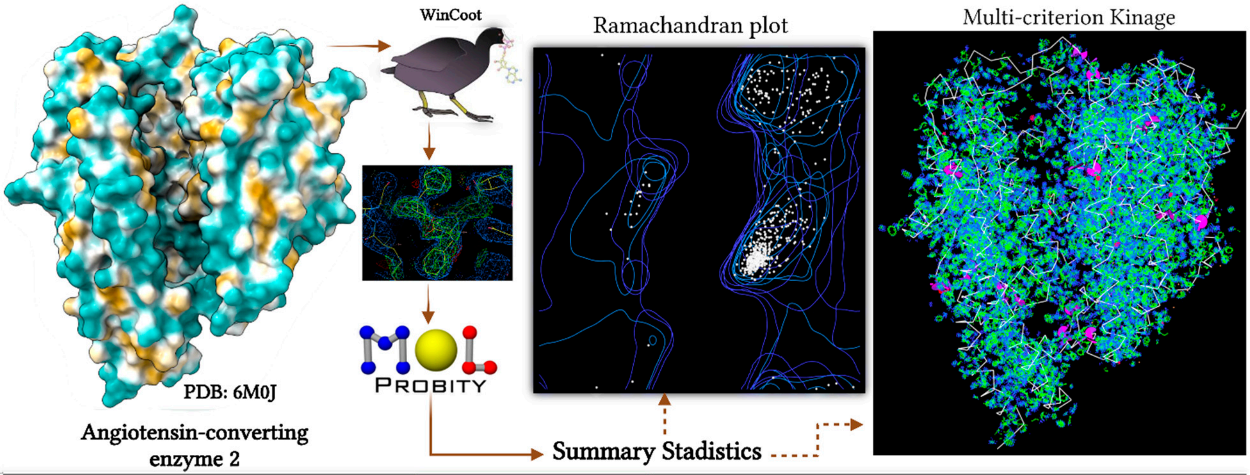

Clashscore, all atoms:	5.14	93rd percentile (N=1784, all resolutions)	
Poor rotamers:	0	0.00%	Goal: <0.3%
Favored rotamers:	518	98.11%	Goal: >98%
Ramachandran outliers:	0	0.00%	Goal: <0.05%
Ramachandran favored:	584	98.15%	Goal: >98%
MolProbity score^:	1.27	99th percentile (N=27675, 0Å - 99Å)	
Bad bonds:	0 / 5016	0.00%	Goal: 0
Bad angles:	0 / 6817	0.00%	Goal: 0%

Figure S2. Network Topology Reveals Key Anchoring Residues in Polar/Charged Fatty Acid-ACE2 Interactions. Complementing the hydrophobic interaction map, this network diagram provides a detailed visualization of the predicted polar and charged interactions stabilizing the fatty acid-ACE2 complexes. Derived from molecular docking simulations (Supplementary Tables S13 - S21), the graph connects fatty acids (central blue nodes) with interacting ACE2 residues (peripheral green nodes) via edges colored according to interaction type: Hydrogen Bonds (red), Salt Bridges (orange), and Carbon-Hydrogen Bonds (gray). The network highlights the critical role of specific polar/charged residues, such as Lys74, Lys441,

and Arg518, which appear to serve as key anchoring points through frequent hydrogen bonding and/or salt bridge formation with multiple fatty acids.

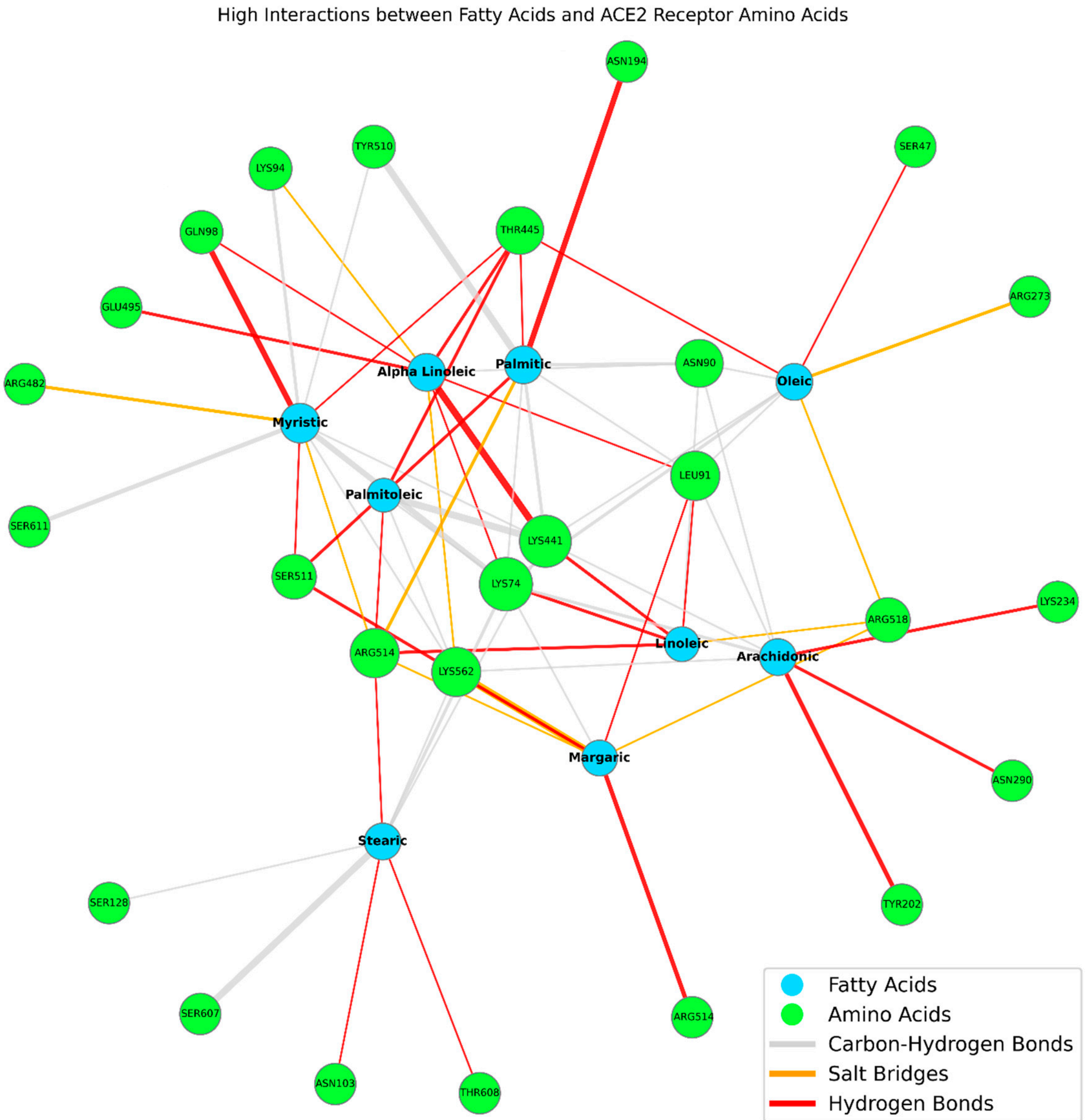

Figure S3. Network Topology Reveals Key Hubs in the Hydrophobic Interaction Landscape of Fatty Acid-ACE2 Binding. This network visualization comprehensively maps the predicted hydrophobic interactions between the nine fatty acids (central blue nodes) and interacting ACE2 amino acid residues (peripheral green nodes), as determined from molecular docking analyses (see Supplementary Tables S2 – S10 for detailed interaction data). Edges represent identified hydrophobic contacts, illustrating the complex web

of nonpolar interactions stabilizing the ligand-receptor complexes. The network highlights residues such as Leu95, Phe390, and Arg393 (note: Arg393 can participate via its aliphatic chain) as prominent hubs, interacting with multiple fatty acids, suggesting their importance in anchoring these ligands within various ACE2 binding regions.

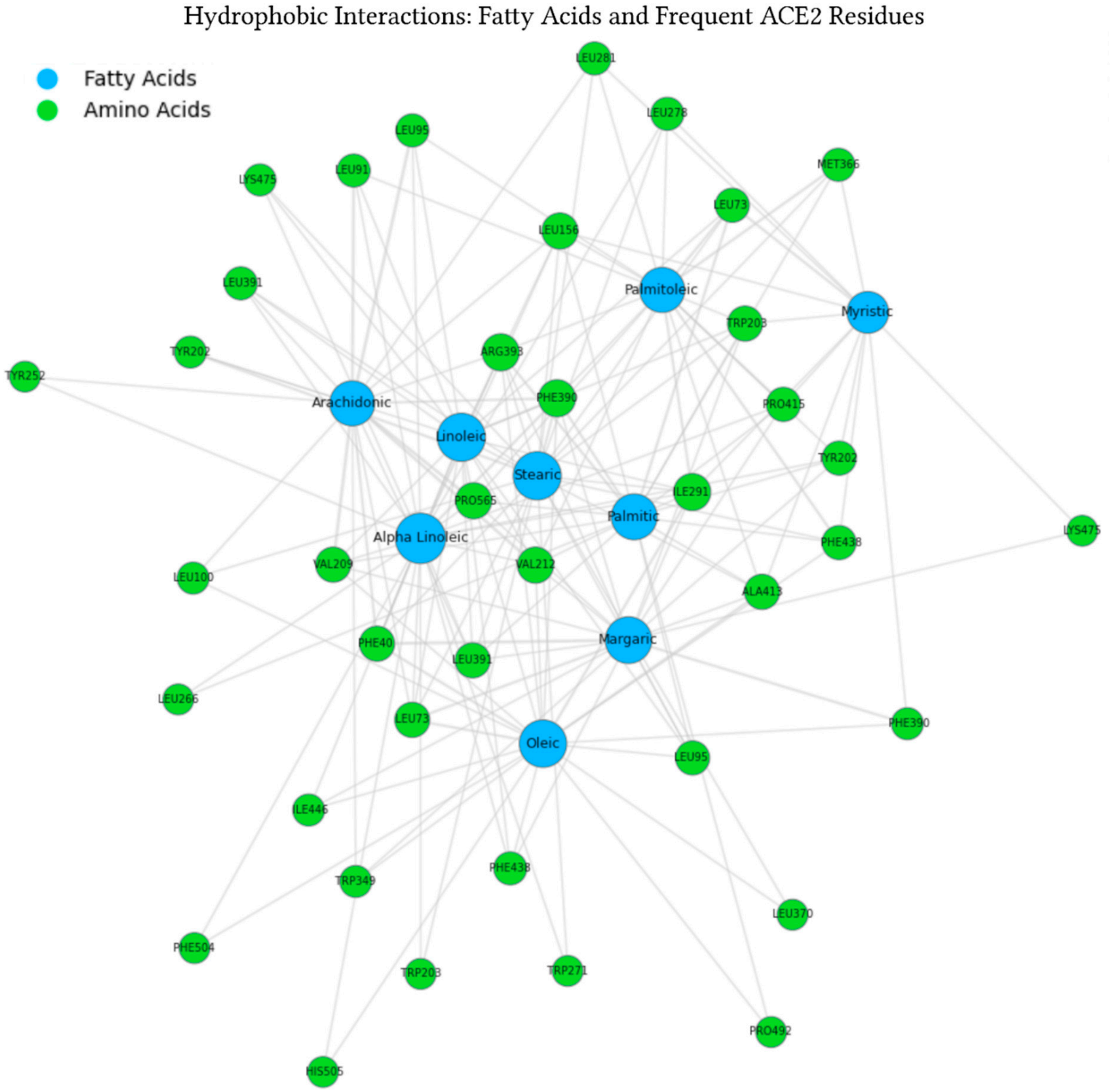

Figure S4. Predicted Binding Mode of Oleic Acid in ACE2 Region 5. Representative highest-affinity docking pose (rank 1/250; SP-dG = –6.97 kcal·mol^{-1}) from SwissDock "Accurate" simulations using the ACE2 structure (PDB: 6M0J; 2.45 Å resolution). (A) Overall ligand–receptor complex showing oleic acid (gray spheres) in the lateral ridge pocket adjacent to the Spike-binding interface. (B) Ligand molecular surface (green mesh) illustrating shape complementarity within the binding cavity. (C) Receptor pocket

surface colored by electrostatic potential, highlighting the hydrophobic cleft. (D) Detailed interactions: salt bridge with ARG518 (2.8 Å, yellow dashed line) and key hydrophobic contacts with ILE291, ALA413, PHE438, and ILE446 (purple dashes).

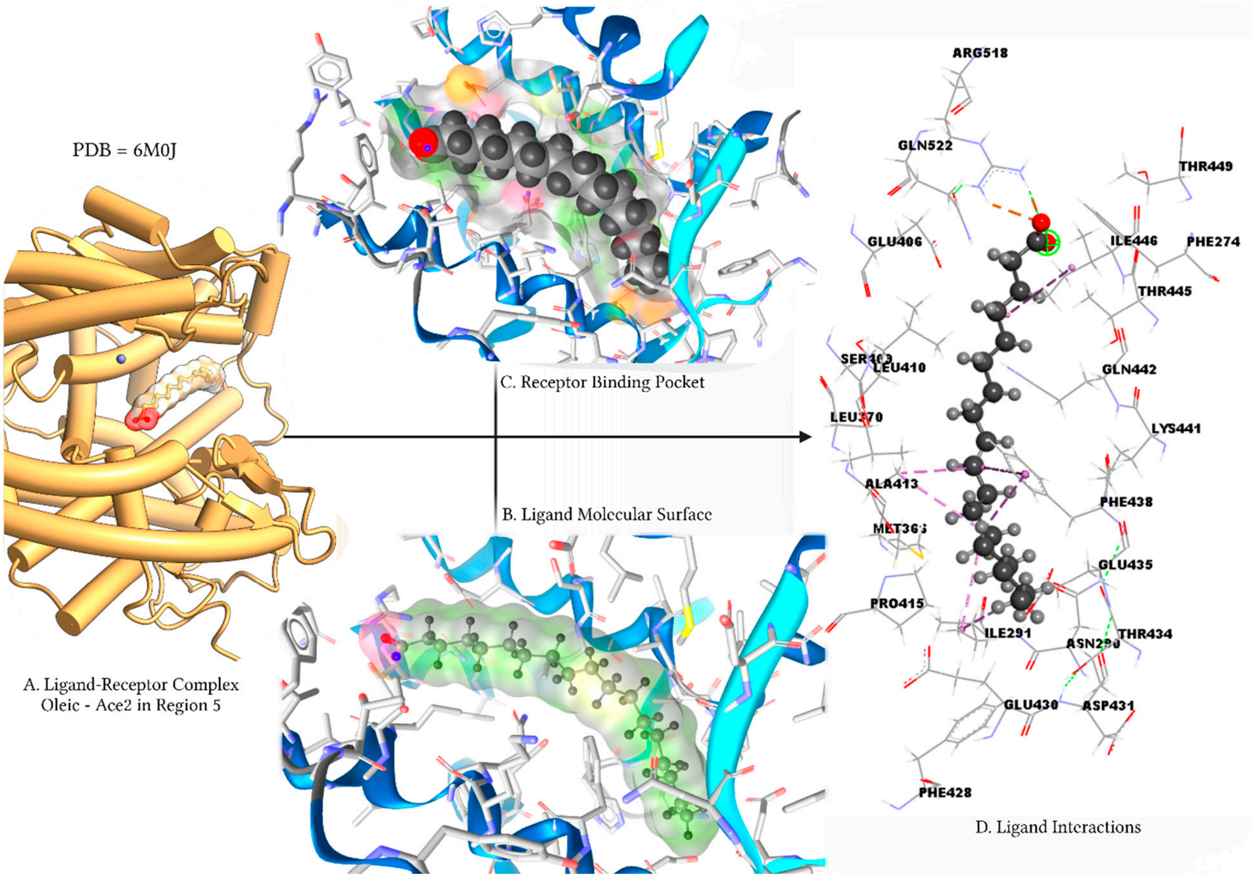

Figure S5. Temporal convergence analysis of MM/PBSA binding free energy calculations. Four-panel convergence assessment showing the temporal evolution of ΔG_{bind} values throughout molecular dynamics simulations for (A) α-linoleic acid, (B) arachidonic acid, (C) linoleic acid, and (D) oleic acid binding to distinct ACE2 receptor regions. Raw trajectory data (translucent lines) are overlaid with adaptive moving averages (bold solid lines) to visualize convergence behavior. Vertical dashed lines, color-coded to match

their respective binding regions (R1, R2, R3, R5, R7), indicate the statistically determined equilibration times using sliding-window Mann-Whitney U tests ($p > 0.05$ threshold). Data to the left of these lines represent the equilibration phase and were excluded from the final binding energy calculations. The gray horizontal reference line denotes $\Delta G_{bind} = 0$ kcal/mol. This analysis validates that all simulations achieved statistical equilibration within the first 30% of the trajectory, ensuring robust free energy estimates. Simulation frames correspond to 1 ps intervals over the 100 ns production phase.

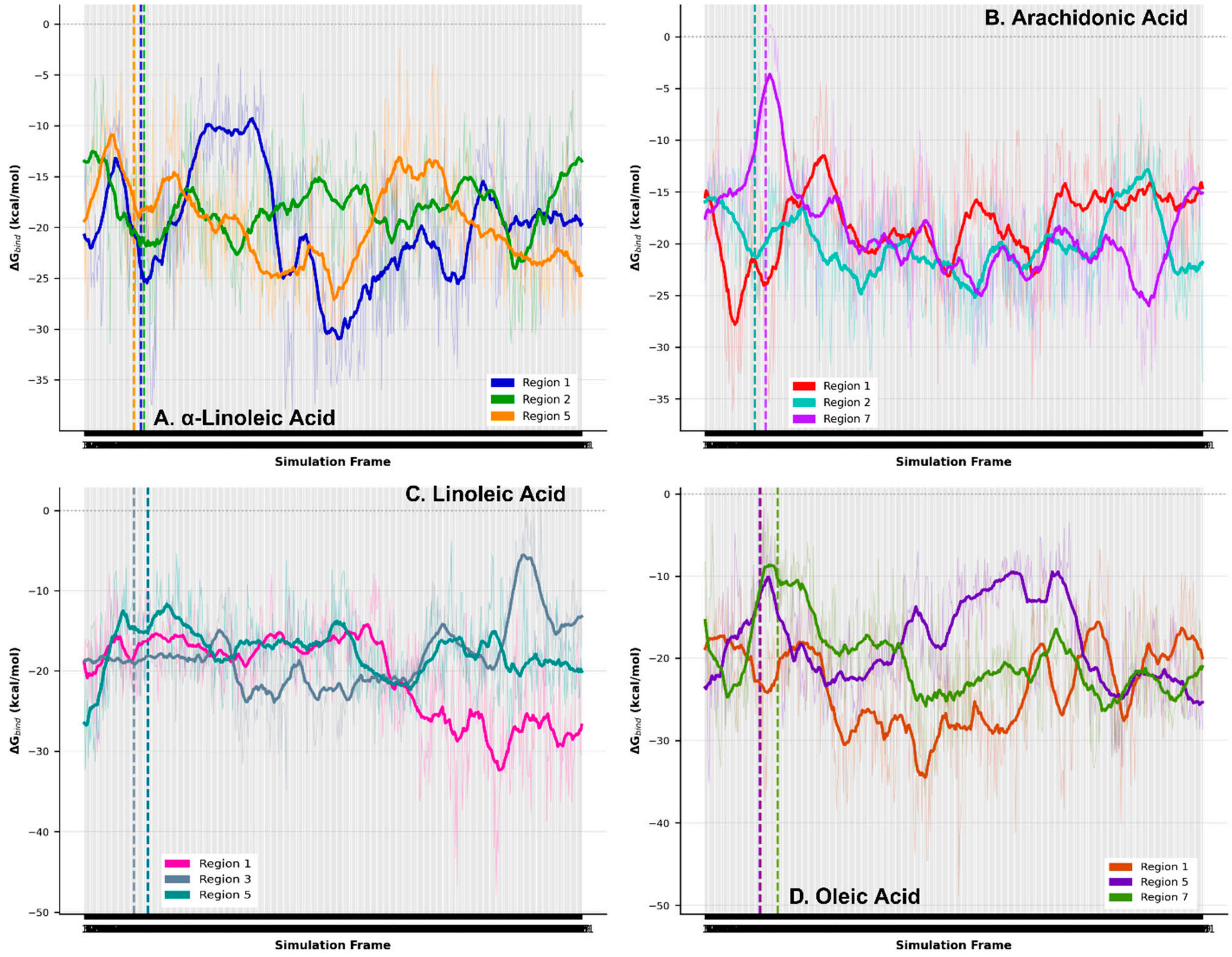

Figure S6. Hierarchical clustering analysis reveals distinct ADMET safety profiles among fatty acids. A multi-dimensional heatmap displays nine fatty acids (rows) across eight critical ADMET parameters (columns) using traffic-light color coding: green (favorable), yellow (intermediate), and red (concerning). Ward linkage clustering was applied to both dimensions using Euclidean distance metrics. Row clustering distinctly separates fatty acid classes: ω-3 (α-linolenic acid) exhibits the most favorable safety profile with minimal hepatotoxicity and mutagenicity risks; ω-6 fatty acids (linoleic, arachidonic) cluster together showing elevated hepatotoxicity and AMES mutagenicity signals; saturated fatty acids (myristic, palmitic, palmitoleic, margaric, stearic) form a coherent group characterized by moderate solubility but excellent safety profiles; and monounsaturated oleic acid occupies an intermediate position. Column clustering reveals three distinct ADMET parameter groups: (i) safety metrics (hepatotoxicity, AMES mutagenicity, hERG inhibition) cluster tightly, indicating correlated toxicity risks; (ii) absorption/distribution parameters

(solubility, intestinal absorption, BBB permeability) separate into a distinct branch reflecting pharmacokinetic properties; and (iii) metabolism markers (CYP3A4 substrate status, P-gp efflux) demonstrate independent clustering patterns. This systematic clustering validates structure-activity relationships in fatty acid ADMET profiles and supports rational prioritization of α-linolenic acid as the lead candidate for dual ACE2/inflammation inhibitor development, combining optimal safety with proven binding affinity. The analysis provides critical insights for experimental validation priorities and formulation strategies in advancing these natural product-derived therapeutics toward clinical translation.

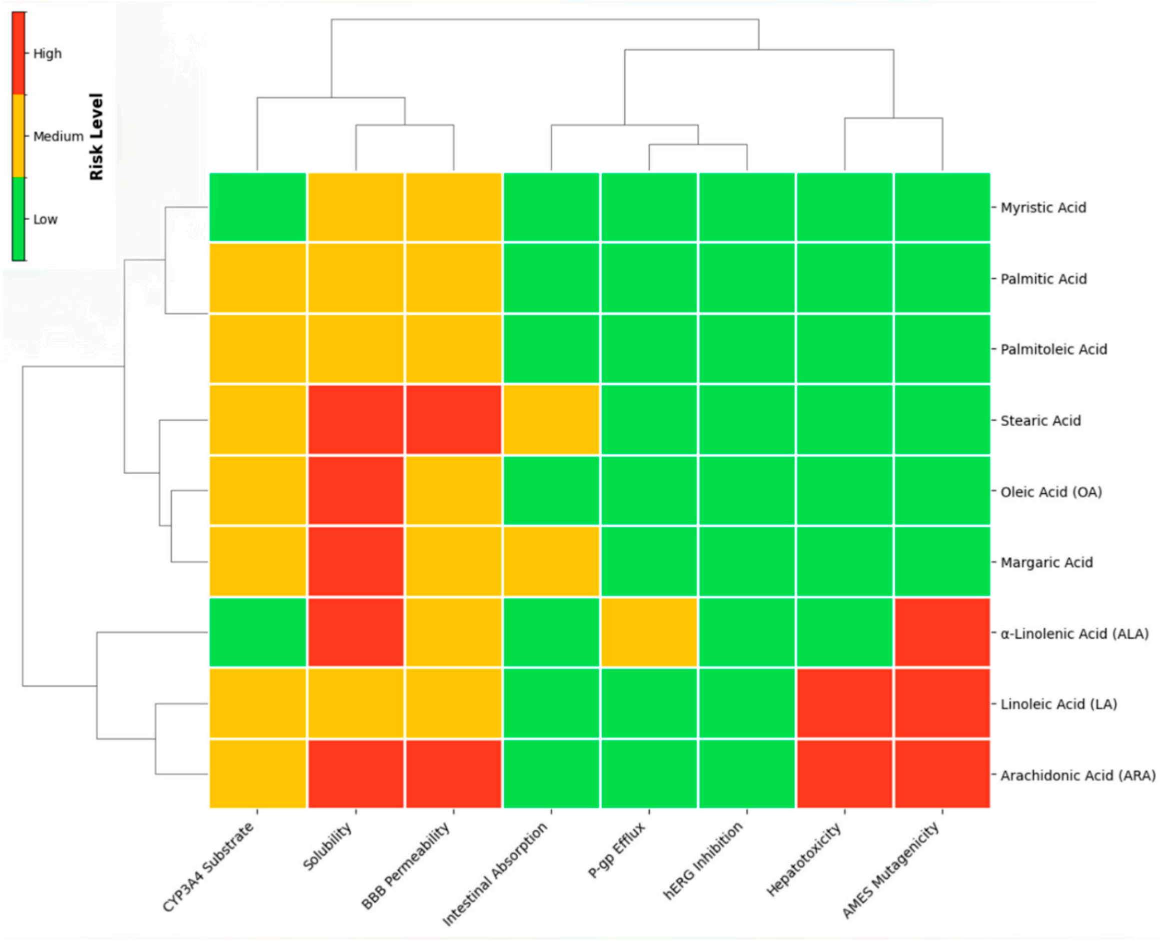

Figure S7. Multi-Platform Activity Heatmap of PassOnline Predictions for Fatty Acids (Pa ≥ 0.90). Each cell represents the predicted activity probability (Pa) of a given fatty acid against key biological targets—ranging from hydrolase and phosphatase inhibitors to CYP substrates and MCSF agonism—on a continuous red–yellow–green scale (0.90–1.00). Fatty acids are grouped atop by structural class (ω-3, ω-6, ω-9, ω-7, saturated) with colored bars and labels matching the inset legend. High Pa values (green) indicate stronger predicted activity. Note the distinct inhibition profiles of saturated versus unsaturated classes and the conserved CYP substrate preferences among ω-3/ω-6 acids, highlighting clear structure–activity relationships across the nine fatty acids.

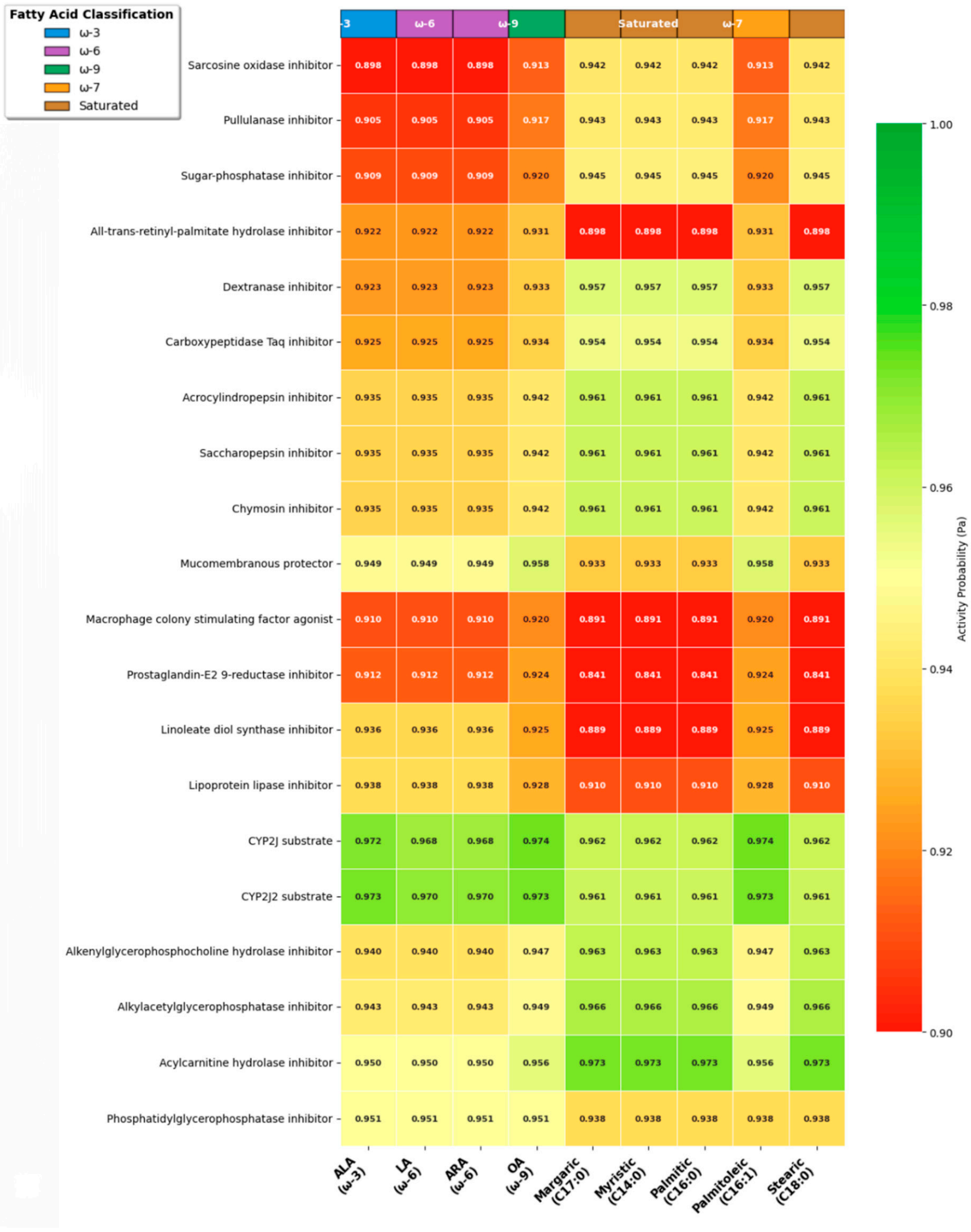
Fatty Acid Classification
ω-3
ω-6
ω-9
ω-7
Saturated
Sarcosine oxidase inhibitor
Pullulanase inhibitor
Sugar-phosphatase inhibitor
All-trans-retinyl-palmitate hydrolase inhibitor
Dextranase inhibitor
Carboxypeptidase Taq inhibitor
Acrocylindropepsin inhibitor
Saccharopepsin inhibitor
Chymosin inhibitor
Mucomembranous protector
Macrophage colony stimulating factor agonist
Prostaglandin-E2 9-reductase inhibitor
Linoleate diol synthase inhibitor
Lipoprotein lipase inhibitor
CYP2J substrate
CYP2J2 substrate
Alkenylglycerophosphocholine hydrolase inhibitor
Alkylacetylglycerophosphatase inhibitor
Acylcarnitine hydrolase inhibitor
Phosphatidylglycerophosphatase inhibitor
ALA (ω-3)
LA (ω-6)
ARA (ω-6)
OA (ω-9)
Margaric (C17:0)
Myristic (C14:0)
Palmitic (C16:0)
Palmitoleic (C16:1)
Stearic (C18:0)
Activity Probability (Pa)
1.00
0.98
0.96
0.94
0.92
0.90

Figure S8. Predicted Interaction Network of Lead Fatty Acids and Key Protein Targets. The network diagram illustrates the polypharmacological profile of four lead fatty acids (central nodes: ALA, LA, ARA, OA) as predicted by PASS Target analysis. Protein targets (peripheral nodes) are connected to fatty acids if the interaction confidence score is >0.3. The thickness of each edge is proportional to the confidence score, indicating the strength of the predicted interaction. Protein targets are color-coded by functional category (Nuclear Receptors, Enzymes, etc.). Edges and nodes highlighted in red signify targets directly related to ACE2 and inflammatory pathways, revealing a dense network of interactions relevant to the dual-action therapeutic hypothesis. The map highlights shared targets, such as 'Oxoeicosanoid receptor 1', and unique interactions, providing a visual guide to the distinct and overlapping mechanisms of each fatty acid.

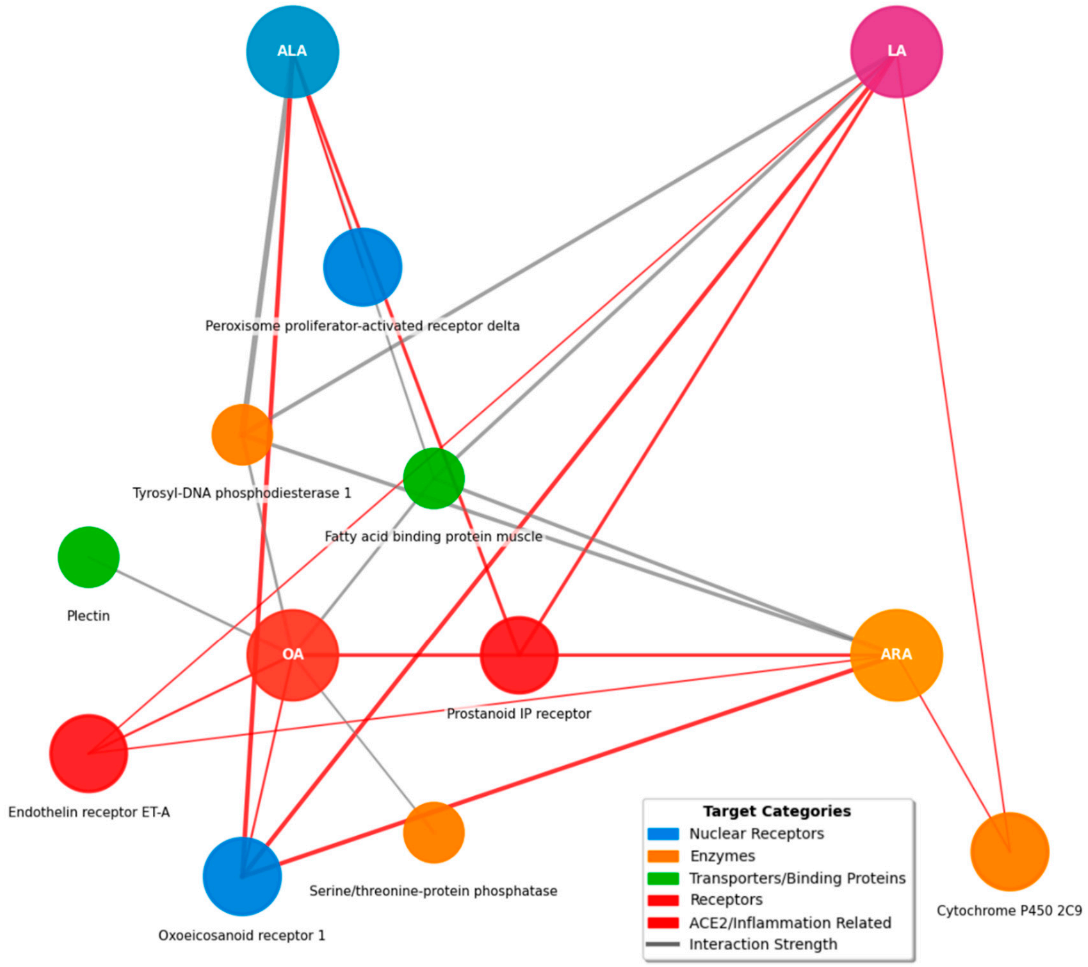

Figure S9. Computational method for the synthesis and molecular docking of fatty acids with the human ACE2 receptor. The process consists of: (1) acquiring ligand (SMILES from PubChem) and receptor (PDB: 6M0J from RCSB) models; (2) optimizing ligand and minimizing energy through Avogadro and converting to mol2 format with Open Babel; (3) preparing the receptor using ChimeraX and WinCoot, validating the structure with MolProbity, and configuring the grid in AutoDockTools; (4) identifying eight binding sites via DoGSiteScorer and information from Reactome, BindingDB, and UniProt; (5) performing flexible docking with SwissDock (EADock DSS, CHARMM) and producing various poses; (6) choosing optimal conformations based on SP-dG and FullFitness assessments, with interaction evaluation in LigandScout, Discovery Studio, Maestro, and PLIP.

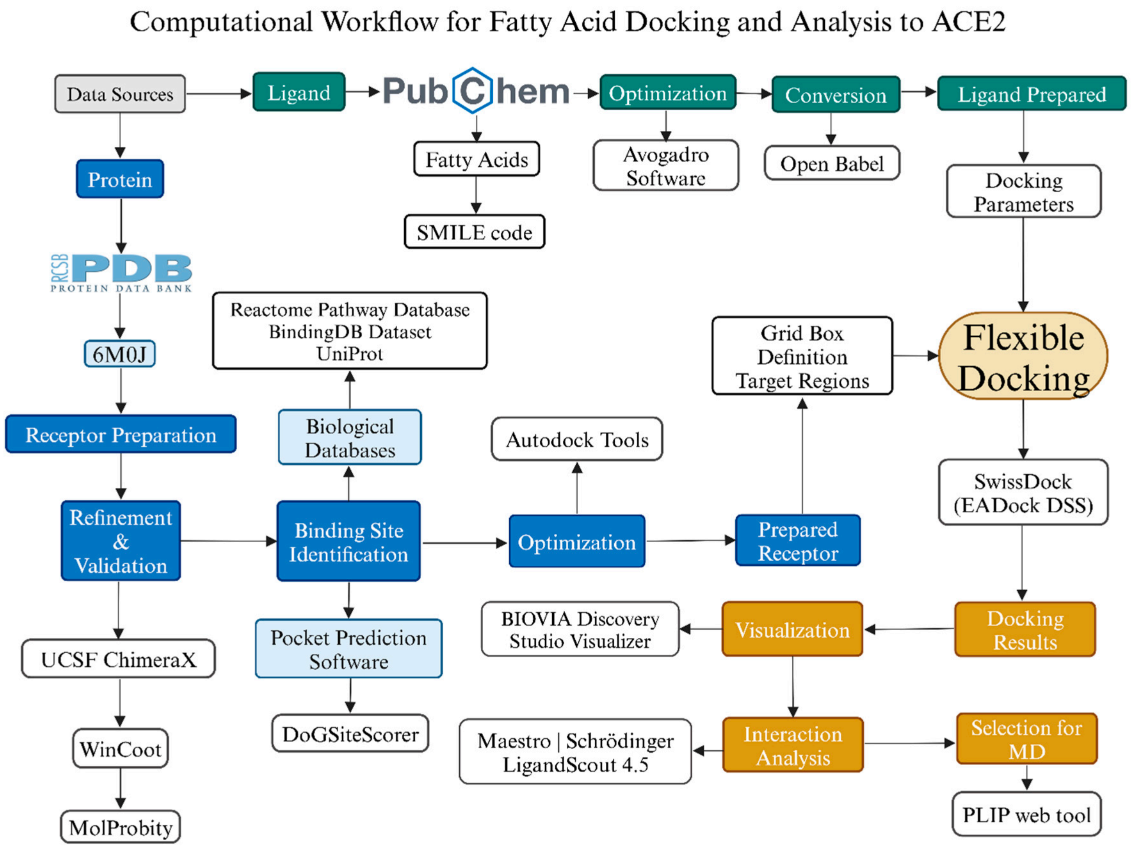

Figure S10. Parameters for molecular docking in specific regions of the ACE2 receptor. Diagram of the eight chosen cavities in ACE2 (PDB 6M0J) showing the precise measurements utilized for the docking experiments. For every region, the following details are provided: (A) the coordinates of the center of the scanning box (X, Y, Z in Å) and (B) the dimensions of the box along the three axes. These values delineated the search space in SwissDock, ensuring that the sampling encompassed both the Spike protein binding site and the allosteric regions implicated in ACE2 dimerization.

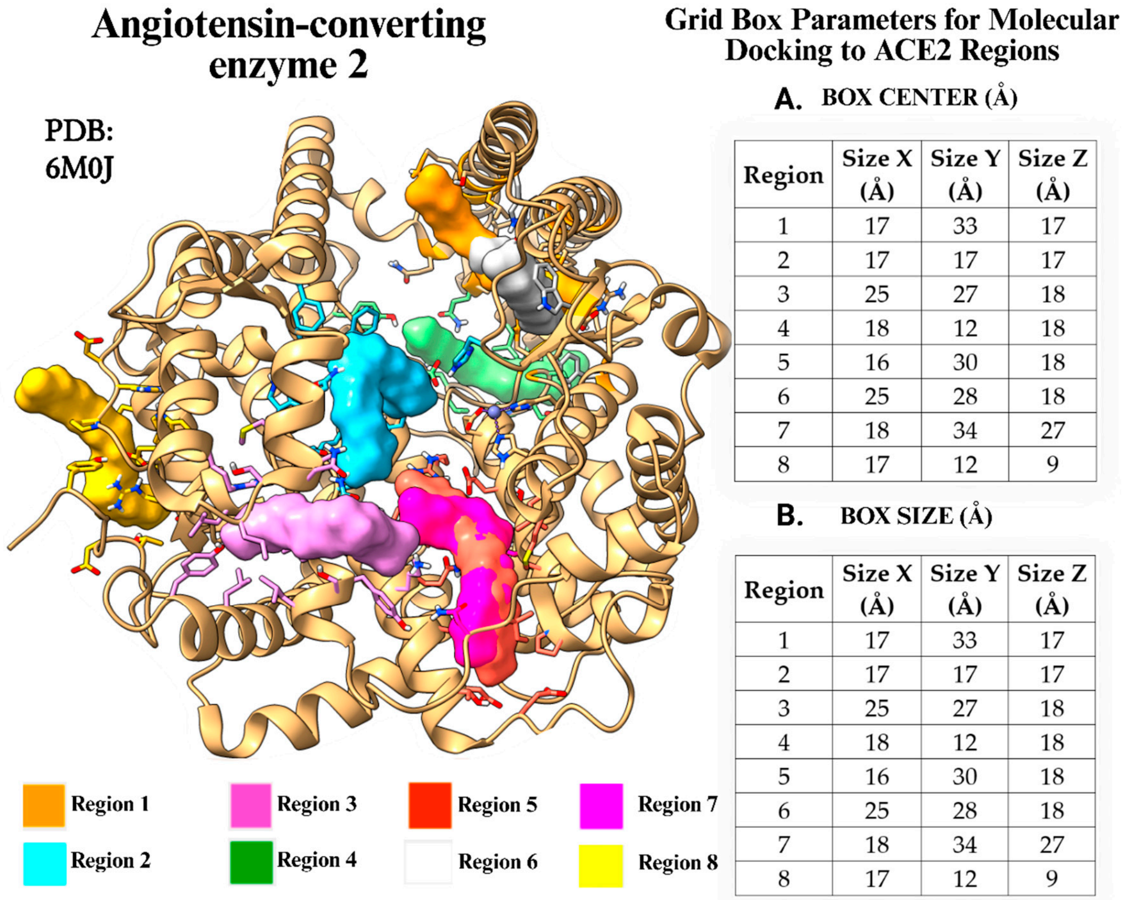

Grid Box Parameters for Molecular Docking to ACE2 Regions

A. BOX CENTER (Å)

Region	Size X (Å)	Size Y (Å)	Size Z (Å)
1	17	33	17
2	17	17	17
3	25	27	18
4	18	12	18
5	16	30	18
6	25	28	18
7	18	34	27
8	17	12	9

B. BOX SIZE (Å)

Region	Size X (Å)	Size Y (Å)	Size Z (Å)
1	17	33	17
2	17	17	17
3	25	27	18
4	18	12	18
5	16	30	18
6	25	28	18
7	18	34	27
8	17	12	9

Figure S11. In silico process for the pharmacological assessment of fatty acids. The illustration outlines the computational steps involved in assessing the therapeutic characteristics of the compounds: (1) The PASS Online tool predicts bioactivity (Pa > 0.70) to detect antiviral, renin-angiotensin system altering, and immune-modulating functions. (2) ADMET assessment utilizing pkCSM, Deep-PK, and SwissADME, enhanced by ADVERPred for toxicity warnings. (3) Combined metabolic forecasting with SOMP (metabolic sites) and SMP (CYP/UGT specificity) to predict biotransformation and possible drug interactions. (4) Mapping molecular targets through KinScreen (inflammatory kinases) and PASS Target (other proteins) creates an interaction landscape that bolsters the idea of dual antiviral-anti-inflammatory effects.

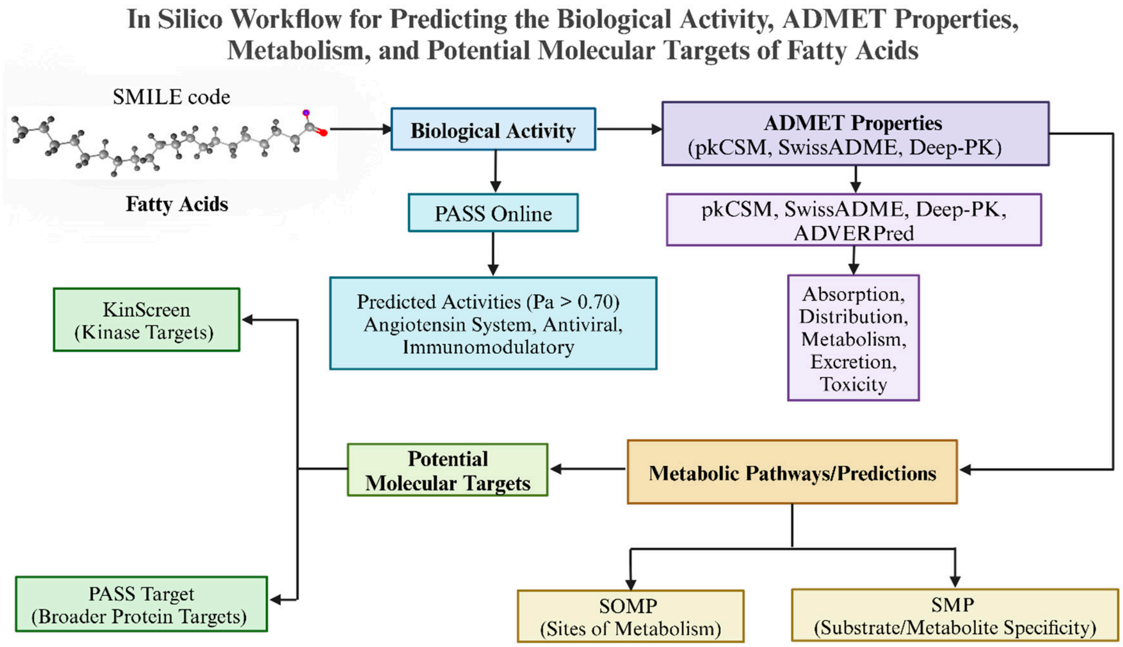

Table S1. Summary of Flexible Molecular Docking Results for Fatty Acids. Data from Table S1 – S9; Figure 7. n = 72 poses total (9 fatty acids × 8 regions). "Best Region" indicates the region with the minimum SP-dG score. Regions defined as in Figure S10. Note: Rows are ordered by descending mean SP-dG. Unsaturated fatty acids (top four) are visually distinguished from saturated (bottom five).

Fatty Acid	Mean SP-dG (kcal/mol) ± SD	Range SP-dG (kcal/mol) (Range = max–min)	Best Score (kcal/mol)	Best Region
**Arachidonic acid**	–6.97 ± 0.21	–7.28 to –6.60 (0.68)	–7.28	Region 7
**Linoleic acid**	–6.92 ± 0.19	–7.25 to –6.73 (0.52)	–7.25	Region 3
**Oleic acid**	–6.76 ± 0.29	–7.12 to –6.24 (0.88)	–7.12	Region 1
**α-Linolenic acid**	–6.74 ± 0.33	–7.11 to –6.12 (0.99)	–7.11	Region 3
**Margaric acid**	–6.75 ± 0.24	–7.08 to –6.39 (0.69)	–7.08	Region 6
**Stearic acid**	–6.73 ± 0.29	–7.20 to –6.21 (0.99)	–7.20	Region 1
**Palmitic acid**	–6.69 ± 0.21	–7.03 to –6.46 (0.57)	–7.03	Region 3
**Palmitoleic acid**	–6.65 ± 0.18	–6.89 to –6.35 (0.54)	–6.89	Region 2
**Myristic acid**	–6.42 ± 0.23	–6.94 to –6.20 (0.74)	–6.94	Region 3

Table S2. Molecular docking results of α-Linoleic Acid with ACE2 Receptor.

**α-LINOLEIC ACID**								
**Region**	**Score**	**AC Score**	**Ligand Scout**	**Hydrophobic Interactions**	**Van der Waals Interactions**	**Carbon-Hydrogen Bonds**	**Hydrogen Bonds**	**Salt Bridges**
**Region 1**	-6,96	-56,69	-23,58	PHE40 - LEU73 - PHE390 - LEU391 - ARG393	PHE32 - SER77 - ALA99 - LEU100 - GLN102 - ASN103 - SER106 - ASP350 - GLY352 - TYR385 - ASN394	-	LYS74	LYS74
**Region 2**	-6,97	-54,65	-25,9	LEU91 - THR92 - LEU95 - VAL209 - VAL212 - PRO565	THR92 - GLN98 - TYR196 - GLY205 - GLU208 - ASN210 - ARG219 - SER563 - GLU564	ASN90	LEU91	-
**Region 3**	-7,11	-56,84	-21,45	LEU156 - TYR252 - LEU266 - LEU278	ALA153 - ASN154 - SER155 - ARG161 - LEU248 - TYR252 - LEU267 - GLY268 - THR276 - ASN277 - TYR279 - SER280 - LEU281	-	LYS441	LYS441
**Region 4**	-6,72	-51,80	-16,39	TYR202 - TRP203 - GLU208	LEU85 - LEU91 - LEU95 - GLN102 - TYR196 - GLY205 - ASP206 - GLU208 - VAL209 - ASN210 - GLU398 - SER511 - ARG514 - LYS562	LYS94	GLN98	LYS94
**Region 5**	-7,01	-52,82	-33,74	ILE291 - PRO415 - GLU435 - PHE438 - LYS441 - HIS540 - LYS541	THR276 - ASN290 - MET366 - LEU370 - ALA413 - THR414 - PHE428 - GLU430 - THR434 - ASN437 - GLN442	-	THR445	LYS441
**Region 6**	-6,46	-54,66	-16,38	PHE40 - PHE390 - LEU391	SER44 - SER43 - SER47 - GLY66 - TRP69 - ALA99 - ARG393 - ASN394	-	-	LYS562
**Region 7**	-6,12	-51,54	-19,33	ASP269 - TRP271 - ARG273 - PHE274 - PHE504 - HIS505	TYR127 - ALA153 - GLY268 - ASP269 - THR276 - ASN277 - TYR279 - LEU503 - PHE504	-	LYS441	LYS441
**Region 8**	-6,61	-55,08	-19,32	LYS475 - ASP494 - GLU495	HIS493 - TRP478 - GLU479 - ARG482 - GLU489 - PRO492 - THR608 - ASP609 - TYR613	-	GLU495	-

Table S3. Molecular Docking Results of Arachidonic Acid with ACE2 Receptor.

ARACHIDONIC ACID								
**Region**	**Score**	**AC Score**	**Ligand Scout**	**Hydrophobic Interactions**	**Van der Waals Interactions**	**Carbon-Hydrogen Bonds**	**Hydrogen Bonds**	**Salt Bridges**
**Region 1**	-7,06	-52,65	-26,68	PHE40 - LEU73 - LEU100 - PHE390 - LEU391 - ARG393	PHE32 - TRP69 - SER70 - SER77 - ALA99 - GLN102 - ASN103 - ASP350 - GLY352 - TYR385 - ASN394	LYS74	LYS74	-
**Region 2**	-7,02	-49,79	-23,33	LEU91 - LEU95 - VAL209 - VAL212 - PRO565	THR92 - GLN98 - GLN102 - TYR196 - GLY205 - ASP206 - GLU208 - ASN210 - SER563 - GLU564	-	TYR202	-
**Region 3**	-6,94	-52,65	-22,50	LEU156 - TYR252 - LEU281	ALA153 - ASN154 - SER155 - ARG161 - LEU248 - LEU266 - GLY268 - ASN277 - LEU278 - TYR279 - SER280 - ASN290	-	LYS441	LYS441
**Region 4**	-6,92	-50,02	-23,29	LEU91 - LEU95 - TYR202 - VAL209 - VAL212 - PRO565	THR92 - GLN98 - GLN102 - TYR196 - TRP203 - GLY205 - ASP206 - GLU208 - ASN210 - SER563 - GLU564	-	-	-
**Region 5**	-6,77	-50,65	-19,96	ALA296 - LEU424 - PRO426	PRO289 - ILE291 - ASP292 - VAL293 - ASP295 - GLN300 - LYS419 - GLY422 - LEU423 - SER425 - PHE428	-	ASN290	-
**Region 6**	-7,14	-53,57	-21,88	PHE40 - TRP349 - PHE390 - LEU391	SER47 - ASN51 - ALA99 - THR347 - ALA348 - ASP350 - ARG393 - ASN394	-	LYS562	LYS562
**Region 7**	-7,28	-43,56	-27,35	LEU91 - LEU95 - VAL209 - VAL212 - PRO565	THR92 - GLN98 - GLY205 - ASP206 - TYR207 - GLU208 - ASN210 - ALA396 - ASN397 - LYS562 - SER563 - GLU564 - TRP566	ASN90	LEU91	-
**Region 8**	-6,60	-44,30	-15,68	TRP478	GLU474 - LYS475 - GLU479 - ARG482 - GLU483 - ILE484 - GLU489 - PRO492 - HIS493 - TRP606 - THR608 - ASP609 - TYR613	-	LYS234	LYS234

Table S4. Molecular Docking Results of Linoleic Acid with ACE2 Receptor.

LINOLEIC ACID								
**Region**	**Score**	**AC Score**	**Ligand Scout**	**Hydrophobic Interactions**	**Van der Waals Interactions**	**Carbon-Hydrogen Bonds**	**Hydrogen Bonds**	**Salt Bridges**
**Region 1**	-7	-63,51	-27,23	LEU73 - PHE390 - LEU391 - ARG393	PHE40 - SER70 - SER77 - ALA99 - LEU100 - GLN102 - ASN103 - SER106 - ASP350 - GLY352 - TYR385 - ASN394	-	LYS74	LYS74
**Region 2**	-6,87	-63,82	-23,96	LEU91 - LEU95 - VAL209 - VAL212 - PRO565	THR92 - GLN98 - TYR196 - GLY205 - ASP206 - GLU208 - ASN210 - SER563 -GLU564	ASN90	LEU91	-
**Region 3**	-7,25	-67,55	-20,72	LEU156 - LEU266	MET152 - ALA153 - ASN154 - SER155 - ARG161 - LEU248 - TYR252 - LEU267 - GLY268 - THR276 - ASN277 - LEU278 - TYR279 - SER280 - LEU281	LYS441	LYS441	-
**Region 4**	-6,73	-64,35	-16,3	TYR202	LEU95 - GLN98 - GLN102 - TYR196 - TRP203 - GLY205 - ASP206 - GLU208 - VAL209 - ASN210 - ALA396 - LYS562 - GLU564 - PRO565 - TRP566	-	-	-
**Region 5**	-6,95	-60,31	-32,01	ILE291 - ALA413 - PHE438 - ILE446	PHE274 - ASN290 - MET366 - LEU370 - GLU406 - SER409 - LEU410 - PRO415 - LEU418 - PHE428 - GLU430 - THR434 - GLU435 - GLN442 - THR445 - THR449 - THR519 - GLN522	-	-	ARG518
**Region 6**	-7,1	66,53	-23,05	PHE40 - LEU73 - ALA99 - PHE390 - LEU391	SER77 - LEU100 - GLN102 - ASN103 - SER106 - ASP350 - LEU351 - GLY352 - ARG393 - ASN394	LYS74	LYS74	LYS74
**Region 7**	-6,73	-62,48	-20,91	LEU95 - TYR202 - TRP203	GLN102 - TYR196 - GLY205 - ASP206 - GLU208 - VAL209 - ALA396 - GLU398 - TYR510 - SER511 - LYS562 - GLU564 - PRO565 - TRP566	-	ARG514	ARG514
**Region 8**	-6,77	-66,8	-17,2	LYS475 - ASP494	MET474 - TRP478 - GLU479 - ARG482 - GLU489 - PRO492 - HIS493 - GLU495 - THR496 - THR608 - ASP609 - TYR613	-	-	-

Table S5. Molecular Docking Results of Margaric Acid with ACE2 Receptor.

MARGARIC ACID								
**Region**	**Score**	**AC Score**	**Ligand Scout**	**Hydrophobic Interactions**	**Van der Waals Interactions**	**Carbon-Hydrogen Bonds**	**Hydrogen Bonds**	**Salt Bridges**
**Region 1**	-7,07	-82	-27	PHE40 - LEU73 - PHE390	TRP69 - SER77 - ALA99 - LEU100 - GLN102 - ASN103 - ASP350 - LEU351 - GLY352 - TYR385 - LEU391 - ARG393 - ASN394	LYS74	-	LYS74
**Region 2**	-6,72	-77,5	-18,91	TYR202 - TRP203	LEU95 - GLN98 -TYR196 - GLY205 - ASP206 - GLU208 - VAL209 - ASN210 - GLU398 - TYR510		SER511 - ARG514	ARG514
**Region 3**	-6,39	-76,6	-27,08	TYR127 - LEU143 - PHE504	SER128 - THR129 - LYS131 - LEU144 - GLU145 - ASN149 -TRP271 - ARG273 - LEU503 - HIS505	-	-	-
**Region 4**	-6,79	-74,3	-26,47	LEU95 - VAL209 - VAL212 - PRO565	ASN90 - THR92 - GLN98 - TYR196 - GLY205 - GLU208 - ASN210 - SER563 - GLU564	LEU91	LEU91	-
**Region 5**	-6,67	-72,8	-29,26	ILE291 - ALA413 - PRO415 - PHE438 - ILE446	PHE274 - ASN290 - MET366 - LEU370 - GLU406 - SER409 - LEU410 - LEU418 - PHE428 - THR434 - GLU435 - GLN442 - THR445 - THR449 - THR519 - GLN522	-	-	ARG518
**Region 6**	-7,08	-82	-27,48	PHE40 - LEU73 - PHE390	TRP69 - SER77 - ALA99 - LEU100 - GLN102 - ASN103 - ASP350 - LEU351 - GLY352 - TYR385 - LEU391 - ARG393 - ASN394	LYS74	-	LYS74
**Region 7**	-6,75	-75,7	-18,34	PHE40 - TRP349 - PHE390 - LEU391 - ARG393	ALA99 - THR347 - ALA348 - ASP350 - ASN394	LYS562	-	LYS562
**Region 8**	-6,54	-74,7	-12,56	LYS475	MET474 - TRP478 - GLU479 - ARG482 - GLU483 - GLU489 - PRO492 - HIS493 - ASP494 - THR608 - ASP609- TYR613	-	-	-

Table S6. Molecular Docking Results of Myristic Acid with ACE2 Receptor.

MYRISTIC ACID								
**Region**	**Score**	**AC Score**	**Ligand Scout**	**Hydrophobic Interactions**	**Van der Waals Interactions**	**Carbon-Hydrogen Bonds**	**Hydrogen Bonds**	**Salt Bridges**
**Region 1**	-6,36	-70,08	-14,89	LEU73 - LYS74	SER70 - SER77 - ALA99 - LEU100 - GLN102 - ASN103 - PHE390 - LEU391 - ASN394	LYS562	-	LYS562
**Region 2**	-6,43	-68,19	-19,06	LEU95	LEU91 - GLN102 - TYR196 - TYR202 - GLY205 - ASP206 - GLU208 - VAL209 - ASN210 - GLY211 - VAL212	-	GLN98	LYS94
**Region 3**	-6,94	-76,94	-21,07	LEU156 - LEU278 - LEU281	ALA153 - ASN154 - SER155 - ARG161 - LEU248 - TYR252 - LEU266 - LEU267 - GLY268 - THR276 - ASN277 - TYR279 - SER280	LYS441	LYS441	LYS441
**Region 4**	-6,27	-70,54	-23,91	TRP203	GLN98 - TYR196 - TYR202 - GLY205 - ASP206 - GLU208 - ASN210 - ASP509	TYR510	SER511	ARG514
**Region 5**	-6,20	-64,23	-29,42	ILE291 - MET366 - ALA413 - PRO415 - PHE438	THR276 - ASN290 - LEU370 - THR414 - LEU418 - PHE428 - GLU430 - ASP431 - THR434 - GLU435 - GLN442	LYS441	THR445	LYS441
**Region 6**	-6,33	-73,04	-19,75	LEU73 - PHE390	PHE40 - TRP69 - SER77 - ALA99 - LEU100 - GLN102 - ASN103 - LEU391 - ARG393 - ASN394	LYS74	LYS74	LYS74
**Region 7**	-6,47	-71,61	-17,33	TYR202 - TRP203	GLN102 - TYR196 - GLY205 - ASP206 - GLU208 - GLU398 - ASP509 - TYR510 -SER511	-	-	ARG514
**Region 8**	-6,36	-68,24	-13,71	LYS475	MET474 - TRP478 - GLU479 - GLU489 - PRO492 - HIS493 - ASP494 - GLU495 - THR608 - ASP609 - TYR613 - ALA614	SER611	-	ARG482

Table S7. Molecular Docking Results of Oleic Acid with ACE2 Receptor.

OLEIC ACID								
**Region**	**Score**	**AC Score**	**Ligand Scout**	**Hydrophobic Interactions**	**Van der Waals Interactions**	**Carbon-Hydrogen Bonds**	**Hydrogen Bonds**	**Salt Bridges**
**Region 1**	-7,12	-75,72	-20,87	PHE40 - LEU73 - LEU100 - PHE390	TRP69 - SER70 - SER77 - ALA99 - GLN102 - ASN103 - ASP350 - GLY352 - TYR385 - LEU391 - ARG393 - ASN394	LYS74	-	LYS74
**Region 2**	-6,24	-67,95	-24,77	TRP271 - PHE504 - HIS505	TYR127 - LEU144 - GLU145 - ASN149 - GLY268 - ASP269 -PHE274 - THR276 - ASN277 - HIS345 - TYR515	-	-	ARG273
**Region 3**	-7,02	-74,54	-19,45	LEU156	MET152 - ALA153 - ASN154 - SER155 - ARG161 - LEU248 - TYR252 - LEU266 - GLY268 - THR276 - ASN277 - LEU278 - TYR279 - SER280 -LEU281	LYS441	LYS441	LYS441
**Region 4**	-6,56	-65,73	-25,57	LEU95 - VAL209 - VAL212 - PRO565	THR92 - GLN98 - TYR196 - GLY205 - ASP206 - GLU208 - ASN210 - ARG219 - SER563 - GLU564	ASN90	LEU91	-
**Region 5**	-6,97	-71,06	-32,43	ILE291 - ALA413 - PHE438 - ILE446	PHE274 - ASN290 - MET366 - LEU370 - GLU406 - SER409 - LEU410 - PRO415 - PHE428 - GLU430 - ASP431 - THR434 - GLU435 - LYS441 - GLN442 - THR445 - THR449 - GLN522	-	-	ARG518
**Region 6**	-6,55	-69,96	-17,98	PHE40 - LEU73 - TRP349 - PHE390 - ARG393	SER44 - TRP69 - ASP350 - LEU351 - TYR385 - LEU391 - ASN394	SER47	SER47	-
**Region 7**	-6,79	-64,26	-29,17	ILE291 - LEU370 - LEU410 - ALA413 - PHE438	THR276 - PRO289 - ASN290 - MET366 - GLU406 - SER409 - PHE428 - THR434 - GLU435 - ASN437 - LYS441 - GLN442 - ILE446	-	THR445	-
**Region 8**	-6,85	-69,48	-15,63	PRO492	LYS475 - TRP478 - GLU479 - ARG482 - GLU483 - GLU489 - HIS493 - ASP494 - THR608 - ASP609 - TYR613	-	-	-

Table S8. Molecular Docking Results of Palmitic Acid with ACE2 Receptor.

PALMITIC ACID								
**Region**	**Score**	**AC Score**	**Ligand Scout**	**Hydrophobic Interactions**	**Van der Waals Interactions**	**Carbon-Hydrogen Bonds**	**Hydrogen Bonds**	**Salt Bridges**
**Region 1**	-6,36	-70,08	-14,89	LEU73 - PHE390 - LEU391 - ARG393	PHE32 - PHE40 - TRP69 - SER77 - ALA99 - LEU100 - GLN102 - ASN103 - ASP350 - GLY352 - TYR385 - ASN394	LYS74	-	LYS74
**Region 2**	-6,43	-68,19	-19,06	LEU95 - VAL209 - VAL212 - PRO565	THR92 - GLN98 - TYR196 - GLU208 - ASN210 - SER563 - GLU564	ASN90	LEU91	-
**Region 3**	-6,94	-76,94	-21,07	LEU156 - LEU266 - LEU278	MET152 - ALA153 - ASN154 - SER155 - ARG161 - LEU248 - TYR252 - LEU267 - GLY268 - THR276 - ASN277 - TYR279 - SER280 - LEU281 - ASN290	-	-	LYS441
**Region 4**	-6,27	-70,54	-23,91	TYR202 - TRP203	LEU95 - GLN98 - GLN102 - TYR196 - GLY205 - ASP206 - GLU208 - VAL209 - ASN210	TYR510	SER511	ARG514
**Region 5**	-6,20	-64,23	-29,42	ILE291 - MET366 - ALA413 - PRO415 - PHE438	THR276 - ASN290 - ASP367 - LEU370 - PHE428 - GLU430 - ASP431 - THR434 - GLU435	LYS441	THR445	LYS441
**Region 6**	-6,33	-73,04	-19,75	LEU73 - PHE390 - ARG393	PHE40 - TRP69 - SER77 - ALA99 - LEU100 - GLN102 - ASN103 - ASP350 - TYR385 - LEU391 - ASN394	LYS74	-	LYS74
**Region 7**	-6,47	-71,61	-17,33	TYR196 - LYS562	LEU95 - GLN98 - GLN102 - HIS195 - GLY205 - ASP206 - GLU208 - VAL209 - ALA396 - ASN397 - GLU564 - PRO565 - TRP566	ASN194	ASN194	-
**Region 8**	-6,36	-68,24	-13,71	PRO492	LYS475 - TRP478 - GLU479 - ARG482 - GLU489 - HIS493 - ASP494 - THR608 - ASP609 - TYR613	-	-	-

Table S9. Molecular Docking Results of Palmitoleic Acid with ACE2 Receptor.

PALMITOLEIC ACID								
**Region**	Score	AC Score	Ligand Scout	Hydrophobic Interactions	Van der Waals Interactions	Carbon-Hydrogen Bonds	Hydrogen Bonds	Salt Bridges
**Region 1**	-6,83	-72,30	-25,23	LEU73 - PHE390 - ARG393	PHE40 - TRP69 - SER77 - ALA99 - LEU100 - GLN102 - ASN103 - ASP350 - GLY352 - TYR385 - LEU391 - ASN394	LYS74	-	LYS74
**Region 2**	-6,89	-67,13	-26,78	LEU91 - LEU95 - VAL212 - PRO565	THR92 - LYS94 - GLN98 - ALA99 - TYR196 - GLY205 - GLU208 - VAL209 - ASN210 - SER563 - GLU564	LYS562	-	LYS562
**Region 3**	-6,80	-68,69	-20,15	LEU156 - LEU278 - LEU281	MET152 - ALA153 - ASN154 - SER155 - ARG161 - LEU248 - TYR252 - LEU266 - LEU267 - GLY268 - THR276 - ASN277 - TYR279 - SER280	LYS441	LYS441	LYS441
**Region 4**	-6,35	-67,07	-18,23	TYR202 - TRP203	LEU95 - GLN98 - GLN102 - TYR196 - GLY205 - ASP206 - GLU208 - VAL209 - ASN210 - GLU398 - TYR510 - SER511 - LYS562	-	ARG514	ARG514
**Region 5**	-6,63	-66,26	-29,51	ILE291 - MET366 - ALA413 - PRO415 - PHE438	THR276 - ASN290 - ASP367 - LEU370 - THR414 - LEU418 - PHE428 - GLU430 - ASP431 - THR434 - GLU435	LYS441	THR445	LYS441
**Region 6**	-6,58	-68,18	-19,05	LEU73 - LYS74 - LEU100	PHE32 - SER70 - SER77 - ALA99 - GLN102 - ASN103 - PHE390 - LEU391 - ASN394	-	LYS562	LYS562
**Region 7**	-6,52	-64,25	-23,62	ILE291 - MET366 - PRO415 - PHE438	ASN290 - ASP292 - THR294 - THR365 - ASP367 - LEU370 - ALA413 - THR414 - THR434 - GLU435 - LYS441 - HIS540	-	-	-
**Region 8**	-6,56	-70,90	-17,26	PRO321 - MET383 - ALA386 - PHE555	VAL318 - GLY319 - LEU320 - LYS353 - GLY354 - PHE356 - ALA384 - ALA387 - GLY551 - GLN552	-	-	-

Table S10. Molecular Docking Results of Stearic Acid with ACE2 Receptor.

STEARIC ACID								
**Region**	**Score**	**AC Score**	**Ligand Scout**	**Hydrophobic Interactions**	**Van der Waals Interactions**	**Carbon-Hydrogen Bonds**	**Hydrogen Bonds**	**Salt Bridges**
**Region 1**	-7,20	-82,53	-21,50	PHE40 - LEU73 - PHE390 - LEU391	TRP69 - SER77 - ALA99 - LEU100 - GLN102 - ASN103 - SER106 - ASP350 - LEU351 - GLY352 - ARG393 - ASN394 - LYS562	LYS74	LYS74	LYS74
**Region 2**	-6,83	-78,31	-22,03	LEU95 - TYR202 - TRP203	LEU91 - LYS94 - GLN98 - TYR196 - GLY205 - ASP206 - GLU208 - VAL209 - ASN210 - GLU398 - TYR510 - SER511	-	ARG514	ARG514
**Region 3**	-6,66	-76,95	-22,21	LEU156 - LEU278 - LEU281	ALA153 - ASN154 - SER155 - ARG161 - LEU248 - TYR252 - LEU266 - LEU267 - GLY268 - ASN277 - TYR279 - SER280 - ASN290	-	LYS441	LYS441
**Region 4**	-6,52	-76,04	-21,78	ALA99 - TYR202 - TRP203 - TYR510	LEU95 - GLN98 - GLN102 - LYS187 - TYR196 - TYR199 - GLY205 - ASP206 - ASN508 - ASP509 - SER511	LYS562	-	-
**Region 5**	-6,21	-72,45	-22,23	ILE291 - MET366 - LEU370 - PHE438	ASN290 - PRO346 - ASP367 - THR371 - ALA413 - PRO415 - THR434 - GLU435 - LYS441	-	-	-
**Region 6**	-6,95	-77,12	-28,04	LEU73 - LEU100 - PHE390 - LEU391 - ARG393	PHE32 - PHE40 - TRP69 - SER70 - SER77 - ALA99 - GLN102 - ASP350 - LEU351 - GLY352 - TYR385 - ASN394	LYS74	LYS74 - ASN103	-
**Region 7**	-6,67	-77,47	-23,48	ALA153 - TRP271 - PHE274	TYR127 - LEU144 - GLU145 - ASN149 - GLY268 - ASP269 - THR276 - ASN277 - THR445 - PHE504	SER128	-	-
**Region 8**	-6,78	-78,92	-20,84	LYS475 - ASP609	ASP471 - MET474 - TRP478 - GLU479 - ARG482 - GLU489 - PRO492 - HIS493 - ASP494 - GLU495 - TYR613	SER607	THR608	-

Table S11. Hierarchical Dynamics Analysis of ACE2-Fatty Acid Complex Stability. The table summarizes key stability and dynamic metrics for the 12 ACE2-fatty acid complexes derived from 100 ns MD simulations. Complexes are categorized into three dynamic classes (Fast, Intermediate, Slow) based on their integrated autocorrelation time (τ_{int}), which quantifies the intrinsic timescale of structural motions. The analysis reveals that while all complexes maintain core structural integrity (RMSD < 3.5 Å), they exhibit a distinct dynamic hierarchy. Fast dynamics (16.7% of systems) are consistent with high binding site plasticity, intermediate dynamics (58.3%) with cooperative conformational processes, and slow dynamics (25.0%) with global rearrangements of potential allosteric significance. Detailed definitions for each metric are provided in the footnotes.

Fatty Acid	ACE2 Region	Dynamic Class[1]	$RMSD_{backbone}$ (Å)[2]	τ_{int} (ns)[3]	N_{eff}[4]	CV[5]	Statistical Quality[6]	Functional Implications[7]
				Fast Dynamics (τ < 50 ns)				
**Oleic**	Region 1	Fast	2.67 ± 0.23	26.4	19	0.085	Good	High plasticity, rapid conformational adjustment
**Arachidonic**	Region 2	Fast	2.16 ± 0.20	31.2	16	0.093	Good	Optimal binding site flexibility
				Intermediate Dynamics (50 ≤ τ ≤ 150 ns)				
**α-Linoleic**	Region 5	Intermediate	2.27 ± 0.31	52.3	9	0.134	Moderate	Balanced stability-flexibility
**α-Linoleic**	Region 2	Intermediate	2.53 ± 0.30	52.6	9	0.120	Moderate	Cooperative domain rearrangements
**Oleic**	Region 5	Intermediate	2.29 ± 0.16	67.0	7	0.070	Moderate	Intermediate allosteric potential
**Linoleic**	Region 1	Intermediate	3.10 ± 0.28	98.1	5	0.090	Moderate	Functional conformational transitions
**Oleic**	Region 7	Intermediate	2.31 ± 0.24	120.1	4	0.103	Limited	Cooperative binding mechanisms
**Linoleic**	Region 3	Intermediate	1.72 ± 0.08	126.1	4	0.049	Limited	Highest structural stability observed
**Linoleic**	Region 5	Intermediate	1.92 ± 0.21	139.0	4	0.108	Limited	Complex multi-domain interactions
				Slow Dynamics (τ > 150 ns)				
**Arachidonic**	Region 7	Slow	2.32 ± 0.22	183.2	3	0.095	Limited	Long-range allosteric networks
**Arachidonic**	Region 1	Slow	3.24 ± 0.25	297.4	2	0.076	Limited	Global conformational adaptations
**α-Linoleic**	Region 1	Slow	3.35 ± 0.21	303.2	2	0.062	Limited	Extensive structural reorganization

Footnotes:

[1] Dynamic Class: Classification based on integrated autocorrelation time (τ~int~). Fast: τ < 50 ns (local dynamics); Intermediate: 50 ≤ τ ≤ 150 ns (cooperative motions); Slow: τ > 150 ns (global rearrangements).

[2] $RMSD_{backbone}$: Root-mean-square deviation of protein backbone atoms during final 25% of simulation (mean ± standard deviation, Å).

[3] τ_{int}: Integrated autocorrelation time calculated using normalized autocorrelation function. Represents intrinsic timescale of structural relaxation.

[4] N_{eff}: Effective number of independent samples calculated as simulation length divided by statistical inefficiency ($2\tau_{int}$ + 1).

[5] CV: Coefficient of variation (σ/μ) indicating relative structural stability. Lower values represent higher stability.

[6] Statistical Quality: Assessment based on N_{eff} values. Good: $N_{eff} \geq 15$; Moderate: $5 \leq N_{eff} < 15$; Limited: $N_{eff} < 5$.

[7] Functional Implications: Biological interpretation based on timescale classification and binding region functional roles.

Table S12. Computational ligand dynamics classification.

System	Dynamics Class[1]	τ_{int} (ns)[2]	N_{eff}[3]	ΔG_{bind} (kcal/mol)[4]	P_{bound}[5]	N_{events}[6]	t_{event} (frames)[7]	S_{conf}[8]
Stable Binding Dynamics (20 < τint < 80 ns)								
α-Linoleic-R5	Stable	45.5	11	−7.01	0.003	1	3.0	0.810
Arachidonic-R1	Stable	74.1	7	−7.06	0.001	1	1.0	0.854
Arachidonic-R7	Stable	37.9	13	−7.28	0.004	1	4.0	0.614
Oleic-R1	Stable	32.4	15	−7.12	0.004	2	2.0	0.742
Persistent Binding Dynamics (τint > 80 ns)								
α-Linoleic-R1	Persistent	122.2	4	−6.96	0.002	1	2.0	0.853
α-Linoleic-R2	Persistent	143.0	3	−6.97	0.003	1	3.0	0.853
Arachidonic-R2	Persistent	190.7	3	−7.02	0.001	1	1.0	0.778
Linoleic-R1	Persistent	122.1	4	−7.00	0.001	1	1.0	0.905
Linoleic-R3	Persistent	166.0	3	−7.25	0.001	1	1.0	0.784
Linoleic-R5	Persistent	100.8	5	−6.95	0.007	6	1.2	0.774
Oleic-R5	Persistent	113.3	4	−6.97	0.001	1	1.0	0.849
Oleic-R7	Persistent	113.2	4	−6.79	0.002	2	1.0	0.784
Statistical Summary								
Stable (n=4)	Mean ± SD	47.5±18.5	11.5±3.4	−7.12±0.12	0.003±0.0014	1.3±0.5	2.5±1.3	0.755±0.105
Persistent (n=8)	Mean ± SD	133.9±30.6	3.8±0.7	−6.99±0.13	0.002±0.0021	1.6±1.7	1.4±0.7	0.823±0.049

[1] Dynamics Class: Classification based on the integrated autocorrelation time (τ_{int}). Stable: 20 < τ_{int} < 80 ns, representing systems with intermediate temporal correlations. Persistent: τ_{int} > 80 ns, indicating systems with long-range correlations and extended relaxation timescales.
[2] τ_{int} (ns): Integrated autocorrelation time. Represents the timescale (in ns) over which the system's structural memory persists, indicating the duration of correlated molecular motions.
[3] N_{eff}: Effective number of samples. Quantifies the number of statistically independent conformations sampled in the 100 ns trajectory, calculated as N_{total} / (2 τ_{int} + 1).
[4] ΔG_{bind} (kcal/mol): Binding free energy. Estimated from the molecular docking calculations for the starting pose of each simulation.
[5] P_{bound}: Bound state probability. The fraction of simulation time where the ligand RMSD was maintained at ≤ 2.0 Å from its initial binding pose.
[6] N_{events}: Number of events. Number of discrete unbinding/rebinding events, identified using a hysteresis threshold (binding onset: RMSD ≤ 2.0 Å; termination: RMSD > 3.0 Å for >5 consecutive frames) to prevent overcounting from fluctuations.
[7] t_{event} (frames): Mean event duration. The average duration of individual binding events, expressed in simulation frames (1 frame = 2 ps).
[8] S_{conf}: Conformational entropy. Calculated using Shannon's entropy from the ligand RMSD distribution (20-bin histogram, 0.1-10.0 Å), quantifying the diversity of explored poses.

Table S13. Computational analysis of global ACE2 structural compactness during fatty acid binding. Radius of gyration analysis reveals uniform computational stability patterns across all 12 complexes (mean $\tau_{Rg} = 9.3 \pm 4.7$ ns, $CV_{Rg} \leq 0.0087$). The homogeneous Fast/Rigid computational dynamics suggest minimal structural perturbation in MD simulations, providing systematic framework for experimental validation through protein stability studies. Systems with rapid computational equilibration ($\tau_{Rg} < 10$ ns) warrant prioritized experimental investigation through biophysical characterization techniques to validate predicted structural preservation and assess actual protein stability under binding conditions.

System	Mean Rg (Å)[1]	CV_{Rg}[2]	τ_{Rg} (ns)[3]	N_{eff}[4]	Dynamic Class[5]	Experimental Priority
**α-Linoleic Acid**						
Region 1	24.94	0.0068	18.1	1	Fast / Rigid	Moderate: Extended correlation analysis
Region 2	24.87	0.0051	5.9	2	Fast / Rigid	High: Rapid equilibration validation
Region 5	25.64	0.0076	7.8	1	Fast / Rigid	High: Intermediate timescale studies
**Arachidonic Acid**						
Region 1	25.67	0.0066	5.4	2	Fast / Rigid	High: Stability assay validation
Region 2	25.48	0.0072	6.2	2	Fast / Rigid	Highest: Rapid equilibration model
Region 7	25.21	0.0071	5.5	2	Fast / Rigid	High: Consistent sampling validation
**Linoleic Acid**						
Region 1	25.12	0.0078	9.6	1	Fast / Rigid	Moderate: Borderline dynamics analysis
Region 3	25.25	0.0075	16.9	1	Fast / Rigid	Low: Extended timescale validation
Region 5	25.50	0.0087	14.1	1	Fast / Rigid	Moderate: Intermediate correlation studies
**Oleic Acid**						
Region 1	25.31	0.0064	2.8	4	Fast / Rigid	Highest: Superior sampling efficiency
Region 5	25.25	0.0084	11.3	1	Fast / Rigid	Moderate: Moderate correlation validation
Region 7	25.23	0.0069	8.1	1	Fast / Rigid	High: Consistent equilibration studies

Footnotes

[1] Mean Rg (Å): The average Radius of Gyration over the 100 ns trajectory, indicating the mean global compactness of the protein.

[2] CV_{Rg}: The Coefficient of Variation (standard deviation / mean) of the Rg, a unitless measure of the magnitude of compactness fluctuations. Lower values indicate higher rigidity.

[3] τ_{Rg} (ns): The integrated autocorrelation time of the Rg trajectory. Represents the timescale of the global compactness fluctuations.

[4] N_{eff}: The effective number of independent samples in the trajectory, indicating the statistical quality of the sampling for this metric.

[5] Dynamic Class: Classification based on the observed dynamics. All systems fall into a single class characterized by rapid stabilization ("Fast") and low-magnitude fluctuations ("Rigid").

Table S14. Computational analysis of ACE2 surface accessibility dynamics during fatty acid binding.

System	Surface Regime[1]	τ (ns)[2]	N_{eff} [3]	Mean SASA (Å²)[4]	Binding Affinity (kcal/ mol)[5]	Surface Flexibility (CV)[6]
**Arachidonic-R2**	Compact Surface	23.3	21	28,159	–7.02	0.010
**Oleic-R1**	Dynamic Surface	50.9	10	28,111	–7.12	0.010
**α-Linoleic-R5**	Dynamic Surface	59.7	8	28,512	–7.01	0.011
**Oleic-R7**	Dynamic Surface	60.0	8	28,032	–6.79	0.011
**Linoleic-R5**	Dynamic Surface	66.0	8	27,941	–6.95	0.013
**Oleic-R5**	Accessible Surface	96.6	5	27,792	–6.97	0.011
**α-Linoleic-R1**	Accessible Surface	122.9	4	27,704	–6.96	0.013
**Arachidonic-R7**	Accessible Surface	125.5	4	28,196	–7.28	0.012
**Linoleic-R3**	Accessible Surface	132.6	4	27,698	–7.25	0.011
**Linoleic-R1**	Accessible Surface	175.5	3	27,963	–7.00	0.010
**α-Linoleic-R2**	Accessible Surface	176.3	3	27,710	–6.97	0.009
**Arachidonic-R1**	Accessible Surface	215.6	2	29,238	–7.06	0.010

[1] Surface Regime: Classification based on SASA autocorrelation timescales (τ): Compact (<25 ns), Dynamic (25-80 ns), Accessible (>80 ns).
[2] τ (ns): Integrated autocorrelation time indicating surface accessibility dynamics timescales.
[3] N_{eff}: Effective sampling for statistical reliability of computational predictions.
[4] Mean SASA (Å²): Time-averaged solvent accessible surface area over 100 ns.
[5] Binding Affinity (kcal/mol): MM/PBSA binding free energies from Section 2.3.1.
[6] Surface Flexibility (CV): Surface fluctuation magnitude indicating temporal classification basis.

Table S15. Computational analysis of hydrogen bond dynamics patterns during fatty acid binding to ACE2. Quantitative analysis reveals moderate computational polar interactions (global mean: 1.124 ± 0.533 H-bonds per frame) with high temporal variability (CV: 0.956 ± 0.231) suggesting dynamic computational networks.

System	Mean HB (±SD)[1]	CV[2]	Occupancy (%)[3]	τ_{HB}[4]	N_{eff}	Dynamics class[5]
**α-Linoleic – Region 1**	1.727 ± 1.499	0.868	72.3	94.32	5.31	Stable H-bonds
**α-Linoleic – Region 2**	0.618 ± 0.854	1.381	41.8	15.67	31.93	Fast H-bonds
**α-Linoleic – Region 5**	1.179 ± 0.770	0.653	81.0	24.56	20.38	Fast H-bonds
**Arachidonic – Region 1**	0.935 ± 1.100	1.176	55.9	51.36	9.75	Stable H-bonds
**Arachidonic – Region 2**	0.751 ± 0.745	0.991	59.1	23.87	20.96	Stable H-bonds
**Arachidonic – Region 7**	0.593 ± 0.638	1.075	51.5	31.25	16.02	Fast H-bonds
**Linoleic – Region 1**	1.554 ± 1.591	1.024	63.5	224.36	2.23	Stable H-bonds
**Linoleic – Region 3**	0.670 ± 0.844	1.259	44.2	56.85	8.80	Stable H-bonds
**Linoleic – Region 5**	0.945 ± 0.697	0.737	75.1	19.53	25.63	Stable H-bonds
**Oleic – Region 1**	2.466 ± 1.610	0.653	86.3	83.73	5.98	Fast H-bonds
**Oleic – Region 5**	0.811 ± 0.761	0.938	62.5	35.19	14.22	Stable H-bonds
**Oleic – Region 7**	1.236 ± 0.881	0.713	78.6	40.75	12.28	Fast H-bonds

[1] Mean HB (±SD): Time-averaged hydrogen bond count per frame.
[2] Coefficient of Variation (CV): Coefficient of variation (σ/μ) quantifying temporal fluctuation magnitude.
[3] Occupancy (%): Percentage of simulation frames maintaining ≥1 hydrogen bond.
[4] Temporal Metrics: τ_{HB} = Integrated autocorrelation time; N_{eff}: Effective independent samples.
[5] Dynamics Classification: Fast H-bonds (τ < 50 frames), Stable H-bonds (50-200 frames) based on temporal correlation analysis.

Table 16. Computational analysis of protein flexibility modulation during fatty acid binding to ACE2. RMSF analysis reveals computational flexibility changes with statistical significance across multiple systems, with Cohen's d effect sizes indicating rigidizing (negative values) and flexibilizing (positive values) effects on protein backbone mobility.

Fatty Acid	Membrane Region	ΔRMSF (Å)[1]	Effect Size (d)[2]	p-value[3]	Dynamic Effect
**Alphalinoleic**	Region 2	-0.280 ± 0.234	-1.193	$< 1\times10^{-10}$	Rigidizing
**Alphalinoleic**	Region 5	-0.116 ± 0.218	-0.532	$< 1\times10^{-10}$	Rigidizing
**Arachidonic**	Region 2	-0.113 ± 0.238	-0.476	$< 1\times10^{-10}$	Rigidizing
**Linoleic**	Region 5	-0.108 ± 0.249	-0.433	$< 1\times10^{-10}$	Rigidizing
**Arachidonic**	Region 7	-0.053 ± 0.304	-0.175	2.2×10^{-5}	Rigidizing
**Linoleic**	Region 1	+0.017 ± 0.454	+0.037	3.6×10^{-1}	Flexibilizing
**Linoleic**	Region 3	+0.027 ± 0.284	+0.094	2.2×10^{-2}	Flexibilizing
**Alphalinoleic**	Region 1	+0.054 ± 0.325	+0.167	5.1×10^{-5}	Flexibilizing
**Oleic**	Region 5	+0.126 ± 0.314	+0.400	$< 1\times10^{-10}$	Flexibilizing
**Arachidonic**	Region 1	+0.156 ± 0.321	+0.485	$< 1\times10^{-10}$	Flexibilizing
**Oleic**	Region 7	+0.136 ± 0.254	+0.534	$< 1\times10^{-10}$	Flexibilizing
**Oleic**	Region 1	+0.155 ± 0.250	+0.620	$< 1\times10^{-10}$	Flexibilizing

[1] ΔRMSF (Å): Mean flexibility change relative to ligand-free baseline across 597 ACE2 Cα atoms. Negative: rigidizing; positive: flexibilizing.

[2] Effect Size (d): Cohen's d standardized effect magnitude. Classification: $|d| \geq 0.5$ (large), $0.3 \leq |d| < 0.5$ (medium), $|d| < 0.3$ (small).

[3] p-value: One-sample t-test against null hypothesis of zero change.

Table S17. Comprehensive Statistical Summary of MM/PBSA Analyses. This table provides an integrated summary of the statistical analyses for the 12 ACE2–fatty acid complexes over 100 ns. The data includes post-equilibration summary statistics, bootstrap confidence intervals, normality diagnostics, and a summary of the Principal Component Analysis (PCA) of the energetic components. A global Kruskal-Wallis's test confirmed highly significant differences among the systems for all energy components ($p < 1x10^{-35}$ for all).

System	N[a]	Equil. Discarded (%)[b]	ΔG_{bind} (kcal/mol)[c]	95% CI (Bootstrap)[d]	Normality[e]	PCA Drivers (PC1/PC2)[f]	PCA Variance (PC1/PC2)[g]
**Alphalinoleic-R1**	444	11.4%	-20.20 ± 7.51	[-20.91, -19.50]	Non-Normal	EEL/GGAS, VDW/ESURF	64.5%, 26.1%
**Alphalinoleic-R2**	441	12.0%	-18.33 ± 4.68	[-18.78, -17.91]	Non-Normal	EEL/GGAS, VDW/ESURF	64.5%, 26.1%
**Alphalinoleic-R5**	451	10.0%	-20.16 ± 5.46	[-20.68, -19.66]	Non-Normal	EEL/GGAS, VDW/ESURF	64.5%, 26.1%
**Arachidonic-R1**	451	10.0%	-17.75 ± 5.24	[-18.25, -17.28]	Non-Normal	GGAS/EEL, VDW/ESURF	63.3%, 30.8%
**Arachidonic-R2**	451	10.0%	-20.36 ± 4.73	[-20.79, -19.93]	**Normal**	GGAS/EEL, VDW/ESURF	63.3%, 30.8%
**Arachidonic-R7**	440	12.2%	-19.44 ± 6.06	[-20.00, -18.86]	Non-Normal	GGAS/EEL, VDW/ESURF	63.3%, 30.8%
**Linoleic-R1**	437	12.8%	-21.07 ± 7.07	[-21.77, -20.44]	Non-Normal	GGAS/EEL, ESURF/VDW	63.0%, 31.5%
**Linoleic-R3**	451	10.0%	-18.20 ± 5.66	[-18.72, -17.67]	Non-Normal	GGAS/EEL, ESURF/VDW	63.0%, 31.5%
**Linoleic-R5**	437	12.8%	-17.45 ± 4.46	[-17.86, -17.03]	Non-Normal	GGAS/EEL, ESURF/VDW	63.0%, 31.5%
**Oleic-R1**	445	11.2%	-24.12 ± 7.42	[-24.81, -23.42]	Non-Normal	EEL/GGAS, ESURF/VDW	74.1%, 19.5%
**Oleic-R5**	446	11.0%	-17.89 ± 6.46	[-18.50, -17.27]	Non-Normal	EEL/GGAS, ESURF/VDW	74.1%, 19.5%
**Oleic-R7**	428	14.6%	-20.78 ± 5.79	[-21.31, -20.22]	**Normal**	EEL/GGAS, ESURF/VDW	74.1%, 19.5%

[a] **N:** Number of equilibrated frames used for analysis.

[b] **Equil. Discarded (%):** Percentage of initial frames discarded after automatic equilibration detection via sliding-window Mann-Whitney U test.

[c] **ΔG_{bind} (kcal/mol):** Mean ± standard deviation of the binding free energy.

[d] **95% CI (Bootstrap):** Non-parametric 95% bootstrap confidence interval of the mean.

[e] **Normality:** Result of Shapiro-Wilk and Kolmogorov-Smirnov tests (α=0.05). Non-Normal status prompted the use of non-parametric statistics.

[f] **PCA Drivers (PC1/PC2):** The two energy components with the highest absolute loadings for Principal Component 1 and 2, respectively. EEL=Electrostatic, GGAS=Gas-phase, VDW=van der Waals, ESURF=Nonpolar solvation.

[g] **PCA Variance (PC1/PC2):** Percentage of total energetic variance explained by the first two principal components.

Table S18. Comparative Performance of PCA and LDA for Regional Energy Discrimination in MM/PBSA Trajectories. All metrics computed from n = 600 samples per fatty acid (3 regions × 200 frames). Higher values are better for Silhouette and Calinski-Harabasz; lower is better for Davies-Bouldin. Δ% represents improvement of LDA over PCA. Dominant components: GGAS (gas-phase energy), EGB (polar solvation), EEL (electrostatic). Bold indicates mechanistic shift between methods ($p < 0.001$ for all LDA improvements via chi-square test).

**Fatty Acid**	**n**	**Silhouette Score**[a]			**Calinski-Harabasz Index**[b]			**Davies-Bouldin Index**[c]			**Variance Explained (%)**[d]		**Dominant Component**[e]	
		PCA	LDA	Δ%	PCA	LDA	Δ%	PCA	LDA	Δ%	PCA	LDA	PCA	LDA
**α-Linolenic**	600	−0.003	0.056*	+2072	36.64	126.82*	+246	4.18	3.58*	+14	90.5	100.0	GGAS	GGAS
**Arachidonic**	600	−0.006	0.011*	+303	26.41	58.06*	+120	4.93	3.55*	+28	94.1	100.0	GGAS	EGB
**Linoleic**	600	0.041	0.093*	+129	86.89	126.61*	+46	2.99	2.44*	+18	94.6	100.0	GGAS	EGB
**Oleic**	600	0.178	0.279*	+57	493.12	949.50*	+93	10.67	1.63*	+85	93.6	100.0	EEL	GGAS

[a] Silhouette Score: Measures cluster cohesion and separation. Range: −1 (misclassified) to +1 (perfectly separated). Values approaching 0 indicate overlapping clusters. Higher values represent superior regional discrimination. Mean calculated across all samples (n = 600 per fatty acid).

[b] Calinski-Harabasz Index: Ratio of between-cluster to within-cluster variance. Higher values indicate better-defined clusters with compact intra-class structure and strong inter-class separation. Scale-invariant metric suitable for comparing clustering quality across different dimensionality reduction methods.

[c] Davies-Bouldin Index: Average similarity between each cluster and its most similar neighbor. Lower values (minimum = 0) indicate superior clustering with minimal overlap. Δ% represents reduction (positive values indicate improvement, as lower DBI is better).

[d] Variance Explained: For PCA, cumulative variance captured by PC1+PC2. For LDA, total discriminative separability explained by LD1+LD2 (or LD1 alone if only 2 regions, yielding 100% by definition for n_classes−1 components).

[e] Dominant Component: MM/PBSA energy term with maximum absolute loading (PCA) or scaling coefficient (LDA) for first discriminant dimension. GGAS = gas-phase energy (VDWAALS + EEL); EGB = generalized Born polar solvation; EEL = electrostatic interaction energy. Bold indicates shift in dominant discriminant between PCA and LDA, revealing mechanism change from unsupervised variance capture to supervised discrimination.

* Statistically superior to PCA ($p < 0.001$, chi-square goodness-of-fit test comparing classification accuracy against random baseline). All analyses derived from 100 ns post-equilibration MD trajectories (3 ACE2 regions per fatty acid, 200 frames per region, stratified random sampling with seed = 42). Improvement percentages (Δ%) calculated as $(Metric_{LDA} - Metric_{PCA})/|Metric_{PCA}| \times 100$. For Davies-Bouldin Index, positive Δ% indicates reduction (improvement).

Table S19. Comprehensive *in silico* ADMET profiling of fatty acids. Systematic pharmacokinetic and safety assessment of nine fatty acid candidates using an integrated panel of computational platforms (pkCSM, Deep-pk, SwissADME, and specialized predictors). Color coding indicates risk stratification: green (🟢) for favorable/low-risk, yellow (🟡) for moderate/intermediate risk, and red (🔴) for high-risk profiles based on established pharmaceutical thresholds. Footnotes provide detailed definitions and the specific platforms used for each parameter. The analysis reveals distinct structure-activity-toxicity relationships, with ω-3 fatty acid α-linolenic acid (ALA) emerging as the lead candidate due to its optimal safety profile, while ω-6 fatty acids (LA, ARA) show higher potency coupled with elevated toxicity signals. This systematic profiling provides a rational basis for lead candidate prioritization for dual ACE2/inflammation inhibition therapy.

Compound	Solubility[a]	Intestinal Absorption[b]	P-gp Efflux[c]	BBB Permeability[d]	Hepatotoxicity[e]	AMES Mutagenicity[f]	hERG Inhibition[g]	CYP3A4 Substrate[h]	Total Clearance[i]	Bioavailability Score[j]
**α-Linolenic Acid (ALA)**	🔴 Poor	🟢 Excellent	🟡 Mixed	🟡 Moderate	🟢 None	🔴 Conflict	🟢 None	🟢 None	🟡 Moderate	🟢 0.85
**Linoleic Acid (LA)**	🟡 Moderate	🟢 Excellent	🟢 None	🟡 Moderate	🔴 Risk	🔴 Conflict	🟢 None	🟡 Yes	🔴 High	🟢 0.85
**Arachidonic Acid (ARA)**	🔴 Poor	🟢 Excellent	🟢 None	🔴 Low	🔴 Risk	🔴 Conflict	🟢 None	🟡 Yes	🔴 High	🟢 0.85
**Oleic Acid (OA)**	🔴 Poor	🟢 Excellent	🟢 None	🟡 Moderate	🟢 None	🟢 None	🟢 None	🟡 Yes	🟡 Moderate	🟢 0.85
**Margaric Acid (C17:0)**	🔴 Poor	🟡 Good	🟢 None	🟡 Moderate	🟢 None	🟢 None	🟢 None	🟡 Yes	🟡 Moderate	🟢 0.85
**Myristic Acid (C14:0)**	🟡 Moderate	🟢 Excellent	🟢 None	🟡 Moderate	🟢 None	🟢 None	🟢 None	🟢 None	🟡 Moderate	🟢 0.85
**Palmitic Acid (C16:0)**	🟡 Moderate	🟢 Excellent	🟢 None	🟡 Moderate	🟢 None[5]	🟢 None	🟢 None[11]	🟡 Yes	🟡 Moderate	🟢 0.85
**Palmitoleic Acid (C16:1)**	🟡 Moderate	🟢 Excellent	🟢 None	🟡 Moderate	🟢 None	🟢 None	🟢 None	🟡 Yes	🟡 Moderate	🟢 0.85
**Stearic Acid (C18:0)**	🔴 Poor	🟡 Good	🟢 None	🔴 Low	🟢 None	🟢 None	🟢 None	🟡 Yes	🟡 Moderate	🟢 0.85

[a] **Solubility:** pkCSM log S / SwissADME ESOL log S; 🔴 <–5.5, 🟡 –5.0 to –5.5, 🟢 >–5.0.
[b] **Intestinal Absorption:** Human intestinal absorption % (pkCSM) / Deep-pk / SwissADME; 🟢 >90%.
[c] **P-gp Efflux:** Predicted substrate by ≥2 tools (🟢 None), conflict indicates mixed (🟡).
[d] **BBB Permeability:** pkCSM log BB / Deep-pk category / SwissADME; 🔴 <–0.2, 🟡 –0.2 to 0.0, 🟢 >0.
[e] **Hepatotoxicity:** Predicted by pkCSM / Deep-pk; 🔴 positive risk, 🟢 none.
[f] **AMES Mutagenicity:** Consensus non-mutagenic (🟢), conflict (🔴).
[g] **hERG Inhibition:** Consensus non-inhibitor (🟢); "Running" or unknown treated as none.
[h] **CYP3A4 Substrate:** Predicted substrate by ≥2 tools (🟡 Yes), otherwise none (🟢).
[i] **Total Clearance:** pkCSM log ml/min/kg / Deep-pk; 🔴 >2.0, 🟡 1.5–2.0, 🟢 <1.5.
SwissADME bioavailability score; 🟢 ≥0.85.
Incomplete data flagged, pending in vitro validation
